# Gastric Microbiota Dysbiosis and Microbiome-Based Interventions in Chronic Atrophic Gastritis

**DOI:** 10.3390/nu18132165

**Published:** 2026-07-03

**Authors:** Ang Li, Yang He, Bushra Walayat, Aamir Saleem, Jing Zhao, Qian Wang, Xiulin Zhang, Changlong Li, Yinhui Liu, Shuming Lu, Ming Li

**Affiliations:** 1 College of Basic Medical Science, Dalian Medical University, Dalian 116044, China; drang33@outlook.com (A.L.); yhliu_dl@163.com (Y.L.); 2 CapitalBio Technology (Liaoning) Co., Ltd., Dalian 116016, China; heyang_hello@163.com (Y.H.);; 3 School of Nursing, Dalian Medical University, Dalian 116044, China; 4 Beijing Key Laboratory of Cancer Invasion and Metastasis Research, Department of Medical Genetics and Developmental Biology, School of Basic Medical Science, Capital Medical University, Beijing 100069, China; 5 Department of Gastroenterology, First Affiliated Hospital of Dalian Medical University, Dalian 116011, China

**Keywords:** chronic atrophic gastritis, CAG, gastric microbiota/gastric microbiome, probiotics, microbial dysbiosis, Correa cascade, *H. pylori* infection

## Abstract

Chronic atrophic gastritis (CAG) is a pivotal precancerous condition in gastric carcinogenesis, with progression typically following the classic Correa cascade. Although *Helicobacter pylori* (*H. pylori*) infection is widely recognized as the principal etiological factor, the persistence of gastric cancer (GC) risk in a subset of patients after successful eradication suggests that gastric microbiota dysbiosis may also contribute to CAG progression. In recent years, high-throughput sequencing technologies have revealed distinct microbial restructuring in patients with CAG, characterized by decreased microbial diversity, depletion of commensal taxa, and enrichment of opportunistic pathogens. These compositional changes are accompanied by metabolic dysfunction, activation of inflammatory signaling pathways, and disruption of immune homeostasis, which may contribute to a microenvironment permissive for precancerous transformation of the gastric mucosa. Probiotics and related microbiome-based therapeutics, including prebiotics, synbiotics, and postbiotics, have emerged as promising adjunctive strategies for *H. pylori* eradication and disease management. Their beneficial effects are mediated through multiple mechanisms, including remodeling of the microbial community, inhibition of pathogen colonization, modulation of host immune responses, and restoration of mucosal barrier integrity. However, whether these interventions can reverse established atrophic or metaplastic lesions remains unclear. In addition, how strain specificity, dose dependency, and interindividual heterogeneity influence clinical efficacy has yet to be fully elucidated. In this review, we summarize the compositional and functional features of gastric microbiota dysbiosis in patients with CAG, as well as the mechanisms and clinical applications of microbiome-based interventions. We further highlight current limitations in the field and discuss future directions for precision microecological therapies integrating multi-omics approaches, engineered probiotics, and artificial intelligence. These advances may provide a theoretical framework and practical guidance for the diagnosis and management of CAG and the prevention of GC.

## 1. Introduction

Atrophic gastritis (AG) is a chronic inflammatory disorder of the gastric mucosa characterized by loss of native gastric glands, mucosal thinning, and intestinal metaplasia (IM) [1]. Among its clinical forms, chronic atrophic gastritis (CAG) is the most frequently encountered and is most commonly associated with persistent *Helicobacter pylori* (*H. pylori*) infection or autoimmune gastritis [2]. Environmental and lifestyle factors, including high salt intake, tobacco and alcohol consumption, may also increase disease susceptibility [3,4]. As a key node in the Correa carcinogenic cascade, CAG may progress to intestinal metaplasia, dysplasia, and even gastric cancer, making it a core target for primary prevention of gastric cancer. However, existing standard therapies—primarily *H. pylori* eradication and acid suppression—have limited efficacy in reversing established gastric atrophy and intestinal metaplasia. This limitation creates an urgent need to identify new therapeutic targets based on emerging pathogenic mechanisms.

The stomach was long considered a virtually sterile organ, owing to the potent bactericidal activity of gastric acid. However, the advent of high-throughput 16S rRNA gene sequencing and shotgun metagenomics has fundamentally reshaped this view by revealing a low-biomass yet compositionally diverse microbial community within the gastric niche [5]. Consequently, the field has shifted from an *H. pylori*-centered, single-pathogen model to a broader ecological framework that examines how the gastric microbiota contributes to the maintenance or disruption of mucosal immune homeostasis [6].

In recent years, comparative profiling studies and meta-analyses have shown that the gastric microbiota undergoes progressive restructuring along the histological continuum from healthy mucosa to CAG and ultimately to gastric carcinoma, characterized by reduced microbial diversity, depletion of putatively beneficial commensals (e.g., *Rothia* and *Neisseria*), and enrichment of oral-derived taxa such as *Streptococcus*, *Veillonella*, and *Prevotella*, as well as opportunistic pathogens including *Fusobacterium*, particularly under hypochlorhydric and atrophic conditions [7,8,9]. This dysbiotic pattern has been repeatedly observed in CAG and at other precancerous stages along the Correa cascade and may provide important insights into the microbial ecology associated with disease progression.

Importantly, while gastric dysbiosis has traditionally been interpreted as a consequence of disease progression, emerging evidence suggests that it may actively contribute to CAG pathogenesis through functional reprogramming of microbial communities and bidirectional interactions between host and microbiota. Growing evidence further suggests that the stomach is embedded within a dynamic oral–gastric–intestinal microbial network. Bidirectional crosstalk along the gastric–gut axis may contribute to CAG pathogenesis [10], while oral microorganisms continuously enter the stomach through swallowing. Moreover, gastric microbiota is not an isolated ecosystem but is shaped by continuous microbial exchange between the oral cavity and the intestine. Oral swallowing constitutes a major source of oral-derived bacteria in the stomach, including *Fusobacterium nucleatum* and *Porphyromonas gingivalis*. Conversely, CAG-associated hypochlorhydria, impaired gastric motility, and bile reflux may facilitate the retrograde colonization of intestinal microbiota, thereby promoting microbial translocation across organ boundaries. Such cross-organ microbial flow may contribute not only to the enrichment of opportunistic pathogens but also to mucosal injury through metabolic reprogramming, such as nitrosamine production, and activation of innate immune pathways. However, the pathophysiological significance of this microbial translocation remains incompletely defined.

Given the central role of gastrointestinal microbial dysbiosis in CAG, microecological interventions have attracted increasing attention. Current evidence suggests that microbiota-targeted interventions may not only suppress pathogenic microorganisms but also restore depleted commensal taxa and their associated ecological functions. Probiotics and prebiotics have shown potential in alleviating gastric mucosal inflammation and enhancing *H. pylori* eradication efficacy [10]. Preclinical studies support these mechanisms, while recent randomized controlled trials and meta-analyses have demonstrated that probiotics can improve *H. pylori* eradication rates and reduce antibiotic-associated adverse effects [11,12]. In addition, probiotics and prebiotics have shown emerging potential in the management of gastric precancerous lesions [11].

However, whether these interventions can reverse or halt established atrophic and metaplastic lesions and thereby improve long-term clinical outcomes remains unclear, as current evidence remains limited and, in some cases, inconsistent [10,12]. Future studies are also needed to clarify how strain specificity, dose–response relationships, and interindividual heterogeneity jointly influence therapeutic efficacy [13,14]. Beyond conventional probiotic approaches, increasing attention has been directed toward the targeted supplementation of functionally relevant commensal microorganisms and broader microbiota reconstruction strategies aimed at restoring ecological niches and microbial metabolic networks. Nevertheless, most current studies still conceptualize the stomach as an isolated therapeutic target and rarely evaluate intervention outcomes within the integrated framework of oral–gastric–intestinal microbial interactions, which may partially explain the incomplete mechanistic understanding of microbiota-based therapies in CAG.

In this review, we summarize recent advances in the characterization of gastric and gastrointestinal microbial alterations in CAG, the mechanisms linking dysbiosis and cross-organ microbial translocation to disease progression, and the emerging clinical evidence supporting probiotic and prebiotic interventions. By integrating local gastric microbial ecology with oral–gastric–intestinal microbial interactions, we aim to provide a more comprehensive framework for understanding the microbial pathogenesis of CAG and to discuss how these insights may inform microbiota-based diagnosis, precision intervention, and future microbiota reconstruction strategies.

## 2. Methods

This narrative review was conducted through a systematic search of the PubMed/MEDLINE and Web of Science databases, with additional manual screening of reference lists from retrieved articles to identify relevant studies not captured by the primary search. The literature search was initiated in October 2025 and updated in June 2026 prior to submission of the revised manuscript, covering publications from inception through May 2026, with priority given to articles published between 2017 and 2026 to ensure that recent advances were comprehensively represented; seminal earlier studies were included where they provided essential mechanistic or historical context.

The following keywords and Boolean combinations were used: (“chronic atrophic gastritis” OR “atrophic gastritis” OR “gastric atrophy”) AND (“gastric microbiota” OR “gastric microbiome” OR “gastric dysbiosis”); (“*Helicobacter pylori*” OR “*H. pylori*”) AND (“microbiota” OR “dysbiosis” OR “eradication”); (“Correa cascade” OR “intestinal metaplasia” OR “gastric carcinogenesis”) AND (“microbiome” OR “microbial composition”); (“probiotics” OR “prebiotics” OR “synbiotics” OR “postbiotics”) AND (“atrophic gastritis” OR “*H. pylori*” OR “gastric mucosa”); (“gastric-gut axis” OR “oral-gastric microbiota” OR “microbial translocation”) AND (“gastric disease” OR “stomach”); and (“SCFA” OR “short-chain fatty acids” OR “bile acids” OR “NF-κB” OR “NLRP3”) AND (“gastric microbiota” OR “atrophic gastritis”).

Eligible study types included original research articles (randomized controlled trials, observational studies, cohort studies, and experimental studies using cell or animal models), systematic reviews, meta-analyses, and narrative reviews published in peer-reviewed English-language journals. Studies were included if they addressed one or more of the following topics: (1) the composition and functional characteristics of the gastric microbiota in health and disease; (2) mechanisms linking gastric microbial dysbiosis to CAG pathogenesis; (3) the gastric-gut axis and inter-organ microbial interactions; or (4) clinical or preclinical evidence for microecological interventions in CAG or *H. pylori* infection. Studies were excluded if they focused exclusively on non-gastric gastrointestinal diseases without relevance to gastric microecology, were published in languages other than English, or lacked sufficient methodological detail for quality assessment. Preprints were not included.

Titles and abstracts were screened for relevance, and full texts were retrieved for all potentially eligible articles. A total of 219 references were ultimately included in this review.

## 3. Epidemiology and Pathogenesis of CAG

### 3.1. Epidemiology of CAG: Global Burden and Emerging Trends

CAG is a major precancerous lesion of GC and imposes a substantial global disease burden. The number of individuals affected by gastritis and duodenitis worldwide reached 31 million in 2019, representing an increase of 12 million compared to 1990 [15]. Across the sequential stages of gastric precancerous lesions, the global prevalence of AG, IM, and dysplasia, also referred to as intraepithelial neoplasia, has been estimated to be 25.4%, 16.2%, and 2.0%, respectively [16]. CAG affects approximately 25% of the examined population [17]. Recent evidence suggests not only an increasing prevalence of CAG but also a shift toward a younger age at diagnosis. A 10-year retrospective study from southern China reported that the prevalence of CAG increased from 18.78% in 2011–2015 to 22.30% in 2016–2020 [18], accompanied by a parallel increase in the proportion of patients younger than 45 years [15]. Over the same period, the prevalence of autoimmune atrophic gastritis (AAG) increased from 13.57% to 30.11% [18]. Among the etiological factors implicated in CAG, *H. pylori* infection remains the strongest known risk factor and is associated with a 2.4-fold higher risk than that in uninfected individuals [17]. However, some patients remain at risk of malignant transformation even after successful *H. pylori* eradication, indicating that the pathogenesis of CAG involves a complex interplay of multiple factors beyond infection alone. These observations further suggest that additional microbial, immunological, and ecological mechanisms may contribute to disease persistence and progression, highlighting the need to move beyond a pathogen-centric view of CAG pathogenesis.

### 3.2. Pathogenesis of CAG

#### 3.2.1. Etiological Factors and Immune-Inflammatory Mechanisms

The pathogenesis of CAG involves multiple interconnected pathways. In *H. pylori*-associated CAG, the bacterium promotes mucosal atrophy through at least two complementary mechanisms. First, at the level of direct cytotoxicity, the type IV secretion system (T4SS) delivers the virulence factor Cytotoxin-associated gene A (CagA) into host epithelial cells, where it perturbs multiple intracellular signaling pathways and induces proinflammatory responses. Vacuolating cytotoxin A (VacA), in turn, causes cellular vacuolation and disrupts mitochondrial function and cell death pathways, thereby directly impairing epithelial homeostasis and barrier integrity [19]. Second, *H. pylori* urease hydrolyzes urea to generate ammonia, creating a relatively alkaline microenvironment that facilitates bacterial survival and colonization. Under conditions of hypochlorhydria or established mucosal atrophy, the increase in intragastric pH and weakening of the acid barrier further diminish colonization resistance. This permits ectopic colonization and relative enrichment of oral-derived bacteria, such as *Streptococcus* and *Veillonella*, whereas genera with putative protective effects are reduced in abundance, thereby exacerbating gastric mucosal injury [20]. Such dysbiosis may act together with virulence factor-mediated injury to accelerate the atrophic process [20].

Importantly, gastric dysbiosis is increasingly recognized as a consequence of altered microbial exchange within the oral–gastric–intestinal axis rather than an isolated gastric phenomenon. Continuous microbial influx from the oral cavity through swallowing, together with potential retrograde migration of intestinal microorganisms under conditions of hypochlorhydria, impaired gastric motility, or bile reflux, may contribute to the accumulation of non-native microbial populations within the stomach. These translocated microorganisms may further aggravate mucosal injury through inflammatory activation, metabolic reprogramming, and disruption of microbial ecological balance. In addition, bile reflux, long-term proton pump inhibitor (PPI) use, and other factors may further promote atrophic progression by altering the gastric microenvironment and disrupting microbial homeostasis [21,22].

In CAG, although anti-parietal cell antibodies (APCAs) and anti-intrinsic factor antibodies (AIFAs) are important diagnostic markers, the principal driver of oxyntic mucosal destruction is a CD4^+^ Th1-dominant cellular immune response directed against the parietal cell H^+^/K^+^-ATPase. This immune response leads to progressive loss of parietal cells and eventual loss of acid-secretory function. Proinflammatory cytokines, including transforming growth factor-β (TGF-β), tumor necrosis factor-α (TNF-α), and interleukin-6 (IL-6), further amplify inflammation and sustain local immune-mediated tissue injury [21,22].

At the effector stage of inflammation, pathogen-associated molecular patterns (PAMPs) derived from *H. pylori* and microorganisms associated with secondary dysbiosis engage pattern recognition receptors (PRRs), particularly Toll-like receptors (TLRs), thereby triggering and sustaining activation of proinflammatory signaling pathways such as NF-κB [23,24]. Concurrently, inflammatory cell infiltration and epithelial stress are accompanied by increased oxidative stress, including elevated levels of reactive oxygen species (ROS) and reactive nitrogen species (RNS) [25,26], which further aggravate epithelial injury and disrupt glandular repair homeostasis [24]. These pathways are not entirely independent. It has been proposed that molecular mimicry between *H. pylori* antigens and the H^+^/K^+^-ATPase may trigger cross-reactive immune responses in genetically susceptible individuals carrying specific MHC class II haplotypes. Consequently, immune-mediated mucosal injury or an atrophic phenotype may persist even after successful bacterial eradication [27,28]. Beyond these immune-mediated mechanisms, bile reflux, iatrogenic hypochlorhydria associated with long-term PPI use, and the accompanying microecological alterations may, individually or in combination, disrupt mucosal homeostasis and promote atrophic changes [29,30]. Collectively, microbial infection, chronic inflammation, and microecological dysregulation interact to drive progressive deterioration of gastric mucosal structure and function, ultimately promoting the development and progression of premalignant lesions.

#### 3.2.2. Inflammation-Driven Metaplastic Reprogramming and Neoplastic Progression

Regardless of the initiating insult, loss of parietal cells disrupts the homeostatic environment of gastric glands. Mature chief cells undergo transdifferentiation through an orderly reprogramming process known as paligenosis, giving rise to spasmolytic polypeptide-expressing metaplasia (SPEM), which is characterized by high expression of trefoil factor family 2 (TFF2). This metaplastic transition is initiated by the interleukin-13 (IL-13) signaling axis driven by group 2 innate lymphoid cells(ILC2s) and is maintained by M2-polarized macrophages within a chronic inflammatory microenvironment [31,32]. CAG represents a critical precursor lesion in gastric carcinogenesis, with disease progression typically following the classical Correa cascade: chronic non-atrophic gastritis → CAG → IM → dysplasia → invasive adenocarcinoma [33]. At the molecular level, *H. pylori* infection activates signaling pathways involving NF-κB and TLRs, thereby sustaining the production of proinflammatory cytokines, such as interleukin-1β (IL-1β), IL-6, and interleukin-8 (IL-8). The resulting oxidative stress, DNA damage, and epigenetic alterations collectively promote malignant transformation of the gastric epithelium [34,35,36,37]. However, accumulating evidence indicates that a subset of patients continues to exhibit gastric mucosal inflammation and remains at an increased risk of neoplastic progression, even after successful *H. pylori* eradication, suggesting that microbial factors beyond *H. pylori* also contribute to the initiation and progression of CAG [38,39]. Given the established role of CAG as a pivotal precursor to GC, elucidating its pathogenic mechanisms and identifying effective preventive and therapeutic strategies are of considerable public health importance.

## 4. Gastric Microbiota: Composition, Dysbiosis, and Disease

### 4.1. Composition and Function of the Gastric Microbiota

#### 4.1.1. Bacterial Diversity and Community Structure of the Gastric Microbiota

The gastric lumen is traditionally regarded as a virtually sterile environment because of its low pH and continuous acid secretion. The discovery of *H. pylori* in the 1980s was the first major challenge to this view, demonstrating that a specific microorganism could colonize the highly acidic gastric niche and initiate disease. However, at that time, research relied mainly on culture-based methods; therefore, investigations were limited to a small number of cultivable bacterial species. This methodological constraint reinforced the long-standing assumption that the gastric microbial community was extremely sparse [40,41].

The emergence of high-throughput sequencing technologies in the early 21st century, particularly 16S rRNA gene sequencing, has markedly advanced our understanding of gastric microecosystems. These approaches have revealed that the healthy human stomach harbors a low-biomass but taxonomically diverse microbial community that was previously underappreciated [42,43]. The gastric microbiota is primarily dominated by the phyla Firmicutes, Proteobacteria, Bacteroidetes, Actinobacteria, and Fusobacteria, whereas the commonly detected genera include *Lactobacillus*, *Bifidobacterium*, *Helicobacter pylori* (typically at low relative abundance), and *Staphylococcus* [44]. Rather than representing a passive microbial background, these communities are increasingly recognized as functionally relevant components of the gastric ecosystem, potentially contributing to mucosal barrier maintenance, modulation of local immune responses, and region-specific metabolic activities.

#### 4.1.2. The Gastric Mycobiome and Virome: Emerging Components of the Gastric Microecosystem

Continuous advances in molecular technologies along with growing evidence indicate that the gastric microbial ecosystem extends beyond bacteria, with fungi and viruses representing important components of gastric microecology. Regarding the mycobiome, although fungi account for only 0.01–0.1% of the gastrointestinal microbial community, their relatively large cell size and distinct immunomodulatory properties suggest that their role in the gastric microenvironment should not be overlooked [45]. Using internal transcribed spacer (ITS) sequencing, investigators have identified multiple fungal genera in gastric mucosal tissues, including *Candida*, *Aspergillus*, *Alternaria*, and *Malassezia*, suggesting that the stomach may harbor a distinct fungal niche despite its acidic environment [46,47]. Intriguingly, animal studies have reported that, in contrast to bacterial communities, gastric fungal diversity may be relatively preserved or even enriched under highly acidic conditions, suggesting that gastric physicochemical properties may selectively shape fungal colonization and ecological adaptation [48]. Regarding the virome, the gastrointestinal tract is also a major reservoir for human-associated viruses, containing an estimated 10^8^–10^10^ virus-like particles (VLPs) per gram of intestinal contents. Among these, bacteriophages predominate, accounting for approximately 97.7% of the viral population, whereas eukaryotic and archaeal viruses constitute only minor fractions [49]. Recent transcriptomic and metagenomic analyses have detected diverse viral signatures in gastric mucosal samples, including bacteriophages as well as eukaryotic viruses such as human cytomegalovirus (HCMV) and human herpesvirus 6 (HHV-6), suggesting that the gastric virome may represent a previously underappreciated component of mucosal microbial ecology [50,51]. However, the functional roles of the gastric mycobiome and virome in mucosal homeostasis, host immunity, and disease progression remain largely unexplored, and their causal contribution to CAG pathogenesis is still speculative.

#### 4.1.3. Functional Roles of the Commensal Gastric Microbiota

In addition to compositional characteristics, the gastric microbiota exhibits context-dependent ecological and immunometabolic activities that collectively contribute to mucosal homeostasis. At the functional level, commensal microbial communities represent key ecological components that contribute to host microbe homeostasis under physiological conditions [52]. For example, Lactobacillus species can produce lactic acid and bacteriocin-like compounds that contribute to local ecological resistance against colonization by opportunistic pathobionts [53]. In addition, gastric microbial communities may participate in mucosal immune modulation, including regulation of secretory IgA responses, shaping of Treg/Th17 balance, and interaction with pattern recognition receptor pathways such as Toll-like receptors (TLRs), thereby contributing to immune equilibrium within the gastric mucosa [54,55]. Commensal microorganisms locally generate short-chain fatty acids (SCFAs), vitamins, and other bioactive metabolites, which provide energy substrates and cytoprotective signals for the gastric epithelium while also influencing gastric acid secretion and mucosal barrier integrity. However, these effects are highly context-dependent and may vary according to microbial composition and gastric physiological conditions [56,57]. Collectively, the stability of commensal-dominated microbial communities is associated with mucosal homeostasis under physiological conditions, whereas dysbiosis characterized by loss of beneficial taxa and expansion of pathobionts has been linked to increased susceptibility to gastritis, peptic ulcer disease, and gastric carcinogenesis [58,59].

Overall, the gastric microbial community, particularly commensal-dominated bacterial assemblages together with fungal and viral components, should be conceptualized as a dynamic ecological system that contributes to mucosal homeostasis through context-dependent immune, metabolic, and barrier-related functions.

#### 4.1.4. Methodological Challenges and Current Limitations

Despite these advances, several methodological limitations continue to constrain our understanding of the gastric microecosystem. In recent years, research on the gastric microecosystem has achieved substantial progress at the bacterial level; however, studies on the gastric mycobiome and virome remain comparatively limited. This imbalance reflects a combination of methodological challenges, limitations of current sequencing technologies, and barriers related to clinical sampling [60]. Technological limitations have also contributed to this knowledge gap. Before high-throughput sequencing became available, microbiological studies largely depended on conventional culture-based methods. Because most gastric fungi and viruses cannot be stably cultured in vitro, early work focused mainly on a small number of cultivable bacterial species, and fungal and viral components were therefore largely overlooked. Even with metagenomic sequencing, fungal DNA typically represents less than 1% of gastrointestinal samples and is easily masked by host DNA and bacterial reads, which reduces detection sensitivity [60]. Meanwhile, virome research is further hindered by the so-called “viral dark matter” problem, whereby approximately 80–90% of enteric viral sequences cannot be reliably annotated using current reference databases, thereby substantially restricting functional interpretation and ecological analysis [61,62]. This annotation bottleneck is likely more pronounced in the stomach, where viral abundance is intrinsically low and reference database coverage remains insufficient [63]. From the perspective of clinical sampling, the rapid development of intestinal microecology research, particularly studies of colonic microbiota, has been facilitated in part by the ease of fecal sample collection. Fecal sampling is noninvasive, readily repeatable, and provides a practical proxy for luminal microbial composition [64]. In contrast, the characterization of the gastric microbiota relies largely on endoscopic biopsy, an inherently invasive procedure that limits the recruitment of healthy control subjects and introduces the possibility of contamination by oral or esophageal microbiota during instrument passage [65,66]. In addition, biopsy specimens yield only limited amounts of tissue, which often cannot meet the simultaneous requirements of metagenomic, transcriptomic, and viral enrichment workflows needed for integrated multi-omics analyses [67]. Together, these factors constrain large-scale population-based studies of the gastric mycobiome and virome. Because human gastric mucosal specimens are difficult to obtain, animal models have become important tools for overcoming these sampling limitations and enabling direct analysis of gastric mucosal fungi and viruses. For instance, preliminary characterization of gastric mucosal fungal communities has been achieved in piglet and murine models, and the potential roles of trans-kingdom fungal–bacterial interactions in regulating the gastric microenvironment have begun to emerge [48,68].

Taken together, continued advances in ITS sequencing, metagenomics, and viromics, along with methodological improvements in endoscopic sampling and the development of noninvasive surrogate approaches, are expected to facilitate a deeper understanding of how intragastric fungi and viruses contribute to CAG progression and gastric carcinogenesis. Specific pathogenic fungi, such as *Candida albicans*, and oncogenic viruses, such as Epstein–Barr virus(EBV), may serve as novel biomarkers for monitoring CAG progression and as potential targets for therapeutic intervention.

### 4.2. Gastric Microbiota Dysbiosis and Associated Diseases

With the continued development of sequencing technologies, from 16S rRNA gene profiling to metagenomic sequencing and integrated multi-omics approaches, it has become evident that the stomach is not a “sterile organ” in the traditional sense, but rather a complex multi-kingdom ecosystem composed of bacteria, fungi, and viruses [69]. The remodeling of this ecological network is closely associated with a broad spectrum of gastric diseases, and its pathogenic significance can no longer be attributed to any single microorganism. Instead, it reflects a pathological process driven by disruption of overall microecological homeostasis.

#### 4.2.1. Gastric Cancer

During gastric carcinogenesis, dysbiosis of the gastric microbiota exhibits increasingly complex multi-kingdom features. In addition to bacterial dysbiosis, increasing evidence suggests that fungal and viral components also play important roles in gastric carcinogenesis. Beyond *H. pylori*, several metagenomic studies have revealed that aberrant enrichment of non-*Helicobacter* taxa, including *Lactobacillus*, *Streptococcus*, and *Fusobacterium*, is closely associated with metabolic reprogramming of tumor tissues and the establishment of a proinflammatory microenvironment [69,70]. Gastric microbiota also undergoes substantial remodeling in GC, characterized by the enrichment of opportunistic fungi, such as *Candida*, *Malassezia*, and *Cutaneotrichosporon*, along with the relative depletion of commensal fungi normally present in healthy gastric mucosa. This fungal dysbiosis may contribute to tumor progression by modulating mucosal immune responses, exacerbating barrier disruption, and amplifying inflammatory signaling [47]. In addition, alterations in the gastric virome have attracted growing attention. EBV infection is closely linked to approximately 10% of gastric adenocarcinomas and is associated with a distinct DNA hypermethylation phenotype, frequent *PIK3CA* mutations, and overexpression of *PD-L1* and *PD-L2* [71,72]. Changes in bacteriophage community structure may also indirectly shape the tumor-associated microenvironment by altering bacterial community stability and metabolic networks [69]. However, compared with bacteria, functional evidence supporting the roles of fungi and viruses in gastric carcinogenesis remains largely correlative, and the underlying causal mechanisms require further elucidation.

##### The Gastric Mycobiome and Its Associations with Gastric Disease

The limited number of studies available on the gastric mycobiome has already yielded several intriguing observations. Zhong et al. characterized fungal dysbiosis in GC based on ITS sequencing and showed that *Candida albicans* was significantly enriched in gastric carcinoma tissues. Moreover, in this single study, *Candida albicans* enrichment was closely associated with reduced fungal diversity and richness within the stomach and showed preliminary discriminatory potential for distinguishing malignant from normal gastric mucosa [73]; this finding awaits validation in independent cohorts before *Candida albicans* can be considered a candidate fungal biomarker. Using ITS2 sequencing, Zhang et al. further profiled the gastric fungal microbiome in GC tissues, adjacent non-cancerous tissues, and healthy control samples, identifying 10 phyla and 301 genera. Fungal dysbiosis associated with GC was marked by an increased Basidiomycota-to-Ascomycota ratio, expansion of opportunistic fungal pathobionts such as *Cutaneotrichosporon* and *Malassezia*, and depletion of putatively beneficial genera, including *Rhizopus* and *Rhodotorula*. These compositional alterations were significantly associated with intragastric concentrations of both proinflammatory and anti-inflammatory cytokines [47]. Of particular interest, Yan et al. reported a landmark study in *Cell* in 2024, in which large-scale culturomics was used to establish a human Cultured Gut Fungi (CGF) catalog comprising 760 fungal genomes from 48 families and 206 species, including 69 previously undescribed taxa [74]. By integrating this resource with more than 11,000 fecal metagenomes, the authors systematically mapped the gut mycobiome across healthy individuals and multiple disease states, thereby providing a valuable genome resource and methodological framework for future studies of the gastric mycobiome.

##### The Gastric Virome and Its Role in Gastric Carcinogenesis

Compared to the gastric mycobiome, the gastric virome remains even less well characterized, although emerging evidence suggests that it may play an important role in gastric disease. EBV infects gastric mucosal epithelial cells and is associated with approximately 10% of gastric adenocarcinomas; accordingly, it has been classified by the International Agency for Research on Cancer (IARC) as a Group I carcinogen [72]. Approximately 8–10% of gastric adenocarcinomas harbor monoclonal EBV DNA. Integrative molecular analyses by the Cancer Genome Atlas (TCGA) have established EBV-associated gastric cancer (EBVaGC) as a distinct molecular subtype characterized by frequent PIK3CA mutations, extensive DNA hypermethylation, overexpression of JAK2, PD-L1, and PD-L2, and abundant immune cell infiltration. EBVaGC is generally associated with a more favorable prognosis than EBV-negative GC [72,75]. Recent studies have also begun to reveal interactions between EBV infection and the gastric microecosystem. EBV-positive GC tissues exhibit markedly reduced bacterial abundance and diversity, whereas pathobionts such as *Shigella*, *Salmonella*, and *Klebsiella* appear to be relatively enriched, suggesting that EBV infection may remodel the gastric microenvironment and promote a proinflammatory tumor milieu [76]. In addition to eukaryotic viruses, bacteriophages, which constitute the dominant component of the virome, have also attracted increasing attention. In a murine model of *H. pylori*-driven colorectal carcinogenesis, Luo et al. used metagenomic profiling to identify a marked expansion of temperate phages during the early stage of infection. These phages carry virulence-associated genes and infect cancer-associated bacteria (e.g., *Enterococcus faecalis*), pointing to a possible role for phage-bacterium interactions in *H. pylori*-related tumorigenesis [77]. Using metagenomic sequencing, Wang et al. further examined the effects of *H. pylori* eradication therapy on the intestinal virome. They observed sustained contraction of viral communities and enhanced phage-bacterium interactions after antibiotic treatment, and these alterations were not fully recovered at six months after therapy. These findings underscore the long-term impact of gastrointestinal antimicrobial therapy on the virome [78]. A major advance in this field was the publication of the Metagenomic Gut Virus (MGV) catalog by Nayfach et al. in *Nature Microbiology* in 2021 [79]. This resource assembled 189,680 viral genomes from more than 11,800 fecal metagenomes and identified 54,118 candidate viral species, 92% of which were previously undescribed. By substantially expanding the reference database for the gut virome, it has provided an important genomic and methodological foundation for future studies of the gastric virome [79]. Nevertheless, direct and systematic studies of the gastric virome remain scarce. The composition, temporal dynamics, and functional contributions of gastric phage communities during the progression from precancerous lesions to GC therefore require much closer investigation.

#### 4.2.2. Gastric Mucosa-Associated Lymphoid Tissue Lymphoma

In gastric mucosa-associated lymphoid tissue (MALT) lymphoma, chronic *H. pylori* infection remains the best-established etiological factor. However, accumulating evidence indicates that lymphomagenesis is not driven by the pathogen alone, but is also closely associated with persistent microbiota dysbiosis and remodeling of the local immune microenvironment [80,81]. Under chronic inflammatory conditions, sustained antigenic stimulation can induce aberrant clonal expansion of B cells, while disruption of the gastric microbial community may further promote abnormal lymphoid proliferation through modulation of T-cell responses and cytokine signaling networks. This disease process exemplifies immune-related tumorigenesis in the setting of gastric microecological disturbance [80,82].

#### 4.2.3. Other Diseases

In addition to the above conditions, gastric microbiota dysbiosis has also been implicated in functional dyspepsia, gastric ulcer disease, and gastric disorders associated with long-term PPI use. Prolonged acid suppression weakens the gastric acid barrier and facilitates the colonization of the stomach by bacteria, fungi, and even viruses of intestinal origin, which in turn further disrupts the microbial community and sustains a state of low-grade inflammation [83,84]. These observations suggest that gastric microbiota dysbiosis may represent a common pathological basis underlying the development and progression of multiple gastric disorders.

### 4.3. Gastric Microbiota Dysbiosis in CAG

The compositional and mechanistic findings summarized in the following sections should be interpreted in the context of several methodological limitations inherent to the current literature. Most studies investigating gastric microbiota alterations in CAG are cross-sectional in design, which precludes inference of temporal or causal relationships. In addition, sample sizes are often limited, and substantial heterogeneity exists in sampling approaches, including endoscopic biopsy, gastric juice aspiration, and mucosal brushing, which capture distinct microbial fractions and are not directly comparable. Moreover, variability arises from differences in sequencing platforms and bioinformatic pipelines, introducing technical heterogeneity that complicates cross-study integration. Beyond these methodological issues, important confounders such as proton pump inhibitor use, prior antibiotic exposure, dietary patterns, geographic origin, age, smoking, and alcohol consumption are inconsistently controlled across studies.

Therefore, in this review, findings from observational studies are interpreted primarily as associations; mechanistic interpretations are reserved for experimental evidence; and causal statements are limited to longitudinal or interventional studies.

With these caveats in mind, although multiple studies have identified *H. pylori* as the principal etiological factor in CAG [7,33,34], eradication of the organism does not invariably restore ecological homeostasis within the gastric mucosa. Rather than representing only structural loss of glandular tissue caused by *H. pylori* infection, CAG may also be considered a “microecological transition phase” characterized by reduced microbial diversity and upregulation of proinflammatory and procarcinogen metabolic pathways [6,85].

#### 4.3.1. Gastric Microbiota Profiles in CAG

CAG can be regarded as a transitional microecological state between a healthy stomach and GC. Studies have demonstrated that the α-diversity of the intragastric microbiota declines progressively along the pathological continuum from normal gastric mucosa to CAG and ultimately to GC [7,86]. This reduction in α-diversity reflects concomitant decreases in both species richness and evenness within the mucosa-associated microbial community, resulting in a more homogeneous ecosystem with a diminished capacity to resist perturbation and re-establish homeostasis. Beyond indicating a loss of microecological complexity, declining diversity is associated with a broader shift in the mucosal microenvironment toward a state characterized by features of chronic inflammation. Whether this association reflects a causal relationship or parallel responses to shared upstream insults remains to be established.

##### Characteristic Alterations in Microbiota Composition During the CAG Stage

**Expansion of pathobionts and opportunistic pathogens.** During the CAG stage, the relative abundance of *Helicobacter*, predominantly *H. pylori,* increases. Concurrently, as gastric acid secretion declines and the mucosal barrier becomes compromised, acid-tolerant, facultatively anaerobic bacteria and oral-derived opportunistic pathogens expand markedly [87]. Representative taxa include *Streptococcus anginosus* and *Veillonella* within the phylum Firmicutes, as well as *Fusobacterium nucleatum* within the phylum Fusobacteria [88,89]. Notably, *Streptococcus* may account for more than 20% of the intragastric microbiota in patients with CAG, an observation that has been replicated across multiple independent cohorts using endoscopic biopsy sampling [90].

**Depletion of commensal taxa.** Genera that normally predominate in the healthy stomach, such as Rothia (Actinobacteria) and Neisseria, show reduced abundance in CAG [43,91]. The directional change in *Prevotella* is inconsistently reported across studies: several biopsy-based studies have reported its depletion in CAG [7,91], whereas other cohorts, particularly those examining later disease stages or using gastric juice samples, have reported enrichment [92]. This discrepancy likely reflects differences in sampling site, disease stage, and geographic origin, highlighting the influence of methodological context on *Prevotella* abundance profiles.

Collectively, the microecological signature of CAG is characterized by reduced community diversity, loss of commensal taxa, and enrichment of opportunistic pathogens. These compositional features are consistently observed across multiple independent cohorts, though their functional contributions to mucosal inflammation and precancerous transformation vary in the strength of supporting evidence. The cross-study reproducibility of key microbial findings is summarized in Table 1.

**Table 1:** Cross-study reproducibility of major gastric microbial alterations in chronic atrophic gastritis (CAG): consistency assessment across independent cohorts.

Taxon	Direction of Change (CAG/GC vs. Controls)	Cohorts Reporting Consistent Findings	Discrepant or Null Reports	Primary Confounders Driving Between-Study Variability	Evidence Type (Human/Animal/In Vitro)	Predominant Sampling Method	Overall Reproducibility
*Streptococcus*	↑ Enriched along Correa cascade; consistently enriched at CAG, IM, and GC stages	Conti et al. 2021 [7]; Fu et al. 2024 [20]; Miao et al. 2022 [58]; Liu C et al. 2022 meta-analysis [92]; Zhang X et al. 2021 [93]; Coker et al. 2018 [94]	Few; inconsistency mainly at species level	*H. pylori* status; PPI use; geographic origin; disease stage	Human (cross-sectional, multi-cohort); Animal model—causal evidence in mice (Fu et al. 2024 [20])	Endoscopic biopsy	High
*Lactobacillus*	↑ Enriched; progressively elevated across CAG, IM, and GC stages; quantitative data in Table 2	Liu C et al. 2022 meta-analysis [92]; Zhang X et al. 2021 [93]; Coker et al. 2018 [94]; Ferreira et al. 2018 [95]	Some cohorts report no significant change; one meta-analysis showed wide confidence intervals	Dietary habits; antibiotic exposure; *H. pylori* eradication status; geographic origin	Human (cross-sectional); metabolic pathway inferences from predictive profiling only—no direct in vitro or animal validation in gastric tissue	Endoscopic biopsy	Moderate–High
*Veillonella*	↑ Enriched; elevated at CAG/IM stage; remains elevated in GC in most cohorts; quantitative data in Table 2	Liu C et al. 2022 meta-analysis [92]; Coker et al. 2018 [94]; He et al. 2022 [96]; Zhang SM et al. 2024 [97]	Some variability across geographic cohorts	PPI use; geographic origin; sampling site (gastric juice vs. biopsy)	Human (cross-sectional); in vitro co-culture confirms lactate cross-feeding with *Streptococcus*/*Lactobacillus* [97]—in vivo gastric validation lacking	Endoscopic biopsy/gastric juice	Moderate
*Prevotella*	Inconsistent: ↓ Depleted in most biopsy-based CAG studies ↑ Enriched in studies of advanced disease or gastric juice samples	Depletion: Conti et al. 2021 [7]; Tokunaga et al. 2025 [8]; Ma et al. 2025 [87]; Yang et al. 2021 [91]; Enrichment: Liu C et al. 2022 [92]; Coker et al. 2018 [94]	Fundamentally contradictory directional changes across studies	Sampling site (biopsy vs. gastric juice); disease stage; geographic origin; oral microbiota contamination during endoscopy	Human (cross-sectional only); no animal or in vitro mechanistic evidence specific to gastric *Prevotella*	Endoscopic biopsy/gastric juice	Inconsistent
*Bifidobacterium*	↓ Depleted; progressive decline along the Correa cascade; quantitative data in Table 2	Liu C et al. 2022 meta-analysis [92]; Coker et al. 2018 [94]; Jeong et al. 2024 [98]; Huo et al. 2025 [99];	Few discrepant reports	Age; dietary fiber intake; prior antibiotic exposure	Human (cross-sectional, multi-cohort); animal model evidence for anti-tumorigenic effects (xenograft); mechanistic data largely extrapolated from intestinal research	Endoscopic biopsy	Moderate
*Bacteroides*	↓ Depleted in human cohorts; Bacteroidetes phylum reduced by 60–80% at GC stage; quantitative data in Table 2	Ferreira et al. 2018 [95]; Wang Y et al. 2024 [100]; Wexler and Goodman 2017 [101]	INS-GAS mouse model suggests procarcinogenic role—contradicts human cohort data [102]	*H. pylori* status; host genetics; disease stage	Human (cross-sectional); Animal model (INS-GAS mice—contradictory to human data [102]); no in vitro gastric-specific evidence	Endoscopic biopsy	Moderate; animal model data contradictory
*Fusobacterium*	↑Enriched, predominantly at GC stage	Aviles-Jimenez et al. 2014 [90]; Liu C et al. 2022 meta-analysis [92]; Ferreira et al. 2018 [95]	Some geographic variability	Geographic origin; sequencing platform; *H. pylori* status	Human (cross-sectional); limited animal or in vitro gastric-specific mechanistic data	Endoscopic biopsy	Moderate
*Helicobacter pylori*	Nonlinear: ↑ Peak at CAG stage ↓ Declines at IM and GC stages; quantitative data in Table 2	Zhang Z et al. 2025 [103]; Fu Q et al. 2025 [104]; Raza et al. 2025 [105]; multiple independent cohorts	Few; nonlinear pattern consistently replicated	Disease stage; eradication history; CagA virulence status	Human (epidemiological cohorts + RCTs—strongest etiological evidence available); animal models confirm carcinogenic mechanisms	Endoscopic biopsy	High
*Escherichia coli*	↑ Enriched; increases progressively from healthy stomach to GC stage; quantitative data in Table 2	Huo et al. 2025 [99]; Castaño-Rodríguez et al. 2017 [106]; Pienaar et al. 2019 [107];	Limited CAG-specific cohort data; not systematically examined	Gastric pH; antibiotic history; disease stage	Human (cross-sectional, limited cohorts); in vitro evidence for acid survival [107]; retrograde translocation in human CAG not formally demonstrated	Endoscopic biopsy	Low–Moderate
*Rothia*	Bidirectional: ↓ Depleted in H. pylori-positive CAG ↑ Enriched in H. pylori-negative CAG/IM	Sung et al. 2020 [38]; Yang et al. 2021 [91];	Direction entirely dependent on H. pylori infection status	*H. pylori* infection status; oral microbiota contamination during endoscopic sampling	Human (cross-sectional only); no animal or in vitro gastric-specific mechanistic evidence	Endoscopic biopsy	Low

Note: Reproducibility was assessed based on directional consistency of findings across independent cohorts as cited in the main text, and does not constitute a formal systematic review or meta-analysis. Criteria—High: consistent findings across ≥5 independent cohorts or confirmed by meta-analysis; Moderate: consistent in 3–4 cohorts with some discrepant reports; Low: ≤2 cohorts or substantial methodological heterogeneity; Inconsistent: contradictory directional changes with no consensus. Other recognized confounders (age, smoking, alcohol consumption) may further influence microbial composition but have not been systematically evaluated for individual taxa in the available literature. None of the taxa listed has been validated as a clinical biomarker for routine CAG diagnosis; all findings remain at the exploratory stage. Arrow notation: ↑ indicates an increase or enrichment in relative abundance; ↓ indicates a decrease or depletion in relative abundance. Abbreviations: CAG, chronic atrophic gastritis; GC, gastric cancer; IM, intestinal metaplasia; PPI, proton pump inhibitor; RCT, randomized controlled trial.

**Table 2:** Trends in the relative abundance of major gastric microbial taxa along the Correa cascade.

Taxon	Typical Colonization Niche	Healthy → Gastritis	Gastritis → CAG	CAG → IM	IM → Dysplasia	Dysplasia → GC	Representative Quantitative Data (Key Source)	Reference (First Author, Year)
Firmicutes								
*Streptococcus*	Oral-derived opportunistic pathobiont	↑/–	>20%	↑↑↑	↑↑↑	↑↑↑↑	Accounts for >20% of intragastric microbiota in some CAG cohorts [90]	Miao (2022 [58]); Zhang (2021) [93]; Aviles-Jimenez (2014) [90]
*Lactobacillus*	Oral-derived; selectively enriched under hypochlorhydria	↑/–	10.4%	≈10.4%	↑↑↑	11.7%	2.9% (healthy) → 10.4% (CAG/IM) → 11.7% (GC) [94]; additional 2.2-fold increase from CAG to GC in a Chinese cohort [93]	Zhang (2021) [93]; Coker (2018) [94]; Ferreira (2018) [95]
*Veillonella*	Oral-derived opportunistic pathobiont	↑	3.8%	≈3.8%	↑↑↑	↑↑↑/–	0.7% (healthy) → 3.8% (CAG/IM); remains elevated in GC in most cohorts; linked to lactate and propionate metabolic pathways by functional pathway analysis	Coker (2018) [94]; He (2022) [96]; Zhang (2024) [97]
Bacteroidetes								
*Prevotella*	Oral-derived commensal; direction of change contested across studies	—/variable	↑/↓	↑/↓	↑/↓	↑/↓	Inconsistent; see Note † and Table 1. Enrichment reported in gastric juice–inclusive meta-analysis (3–5% in healthy → ~15–20% in GC) [92]; depletion reported in most biopsy-based cohort studies [7,8,87,91]	Liu (2022) [92]; Liu (2024) [86]
*Bacteroides*	Gut commensal; obligate intestinal symbiont	—	5–8%	↓↓	↓↓↓	2–3%	10–15% (healthy) → 5–8% (CAG) → 2–3% (GC); Bacteroidetes phylum overall reduced by 60–80% at GC stage; depletion observed in both H. pylori-positive and -negative GC [95,100]	Wang (2024) [100]; Wexler (2017) [101]; Lertpiriyapong (2014) [102]; Ferreira (2018) [95]
Actinobacteria								
*Bifidobacterium*	Gut-derived commensal with probiotic potential	—	4–6%	↓↓	↓↓↓	1–2%	8–12% (healthy) → 4–6% (CAG) → 1–2% (GC) [92,98]	Liu (2022) [92]; Jeong (2024) [98]; Huo (2025) [99]
Proteobacteria								
*Helicobacter pylori* (*H. pylori*)	Gastric-specific colonizer	↑↑	70–90% (peak)	40–50%	↓↓	10–20% (almost absent)	Detection rate: ~0–50% (healthy/asymptomatic) → 70–90% (CAG, peak) → 40–50% (IM) → 10–20% (GC) [103,104]; CagA^+^ strains: 4–6-fold increased carcinogenic risk [105]	Zhang (2025) [103]; Fu Q (2025) [104]; Raza (2025) [105]
*Escherichia coli* (*E. coli*)	Gut-derived; retrograde colonization under hypochlorhydria	—	3–5%	↑↑	↑↑↑	8–12%	0.5–1% (healthy) → 3–5% (CAG) → 8–12% (GC) [99,108]; intragastric pH rise from ~1.5–2.0 to 4.0–5.0 in CAG creates permissive conditions for retrograde colonization [106,107]	Huo (2025) [99]; Chen Z (2025) [108]; Castaño-Rodríguez N (2017) [106]; Pienaar (2019) [107]
Fusobacteria								
*Fusobacterium*	Oral-derived opportunistic pathobiont	—	↑/–	↑	↑↑	↑↑↑	Predominantly enriched at the GC stage; quantitative abundance data vary across geographic cohorts and sequencing platforms [88,90,92,95]	Li H (2024) [88]; Aviles-Jimenez (2014) [90]; Liu (2022) [92]; Ferreira (2018) [95]

Arrow notation: ↑ modest increase; ↑↑ moderate increase; ↑↑↑ marked increase; ↑↑↑↑ pronounced increase; ↓ modest decrease; ↓↓ moderate decrease; ↓↓↓ marked decrease;—no consistent directional change reported; / discrepant findings across studies. *H. pylori* nonlinear pattern: Detection rate peaks at the CAG stage (~70–90%), then declines progressively as atrophy advances, reaching only 10–20% at the GC stage. This nonlinear trajectory reflects progressive remodeling of the gastric niche—including glandular epithelial loss, intragastric pH elevation, and immune reshaping—which together render the environment increasingly unfavorable for *H. pylori* persistence. † *Prevotella* inconsistency: Direction of change is not consistent across studies. Enrichment has been reported in meta-analyses incorporating gastric juice samples [92]; depletion in most biopsy-based cohort studies [7,8,87,91]. No consensus trajectory can be established from current cross-sectional data. For a full assessment of between-study variability and confounders, see Table 1. Note: Rothia bidirectional pattern: Direction is entirely dependent on *H. pylori* infection status: depleted in *H. pylori*-positive CAG, enriched in *H. pylori*-negative CAG/IM [38,91]. No quantitative cross-stage data are currently available. See Table 1 for details. Abbreviations: CAG, chronic atrophic gastritis; GC, gastric cancer; IM, intestinal metaplasia.

##### Changes in Gastric Microbiota Composition Along the Correa Cascade

In healthy individuals, the intragastric microbiota is dominated by commensal taxa from the phyla Firmicutes, Actinobacteria, Bacteroidetes, and Proteobacteria.

Within Firmicutes, the predominant genera include *Streptococcus*, *Lactobacillus*, and *Veillonella*, most of which are oral in origin and remain at relatively low abundance in the healthy acid-secreting stomach. Under conditions of reduced gastric acidity, these taxa appear to gain a colonization advantage, an observation consistently replicated across multiple independent cohorts. Multiple studies published between 2018 and 2024 have reported the persistent enrichment of *Streptococcus* across the stages of CAG, IM, and GC (*p* < 0.05) [58,93]. Beyond compositional enrichment, *Streptococcus anginosus* has been shown to activate proinflammatory MAPK signaling and promote gastric atrophy and tumorigenesis in murine models [20], providing mechanistic plausibility for a contributory role in disease progression; however, direct causal evidence in humans remains lacking. Coker et al. demonstrated that the relative abundance of *Lactobacillus* increased approximately three- to fourfold along the Correa cascade, rising from 2.9% in healthy individuals to 10.4% in patients with CAG or IM and further to 11.7% in those with GC (*p* < 0.01) [94]. Consistent with this, Shen et al. reported in a Chinese cohort that *Lactobacillus* abundance was significantly elevated at the CAG stage and showed a further 2.2-fold increase with progression to GC (*p* < 0.05), with enrichment positively associated with lactate-related metabolic pathways as annotated in KEGG [93]. These findings are consistent with the view that the hypochlorhydric environment of atrophic mucosa creates permissive conditions for Lactobacillus enrichment [94], and suggest a possible association between this genus and metabolic shifts in the gastric mucosa during disease progression, though direct mechanistic evidence in human gastric tissue remains limited [95]. Coker et al. also found that the relative abundance of *Veillonella* rose from 0.7% in healthy controls to 3.8% in patients with CAG or IM and remained elevated in GC [94]. Functional pathway analyses further linked *Veillonella* abundance to the enrichment of lactate and propionate metabolism, as well as short-chain fatty acid biosynthesis pathways [96]. These observations suggest that *Veillonella* may participate in metabolic cross-feeding with lactate-producing genera such as *Streptococcus* and *Lactobacillus*, thereby contributing to shifts in intragastric energy metabolism along the Correa cascade [97]. This inference is supported by in vitro co-culture data and functional pathway analyses; direct in vivo validation in human gastric tissue is currently lacking.

Actinobacteria are mainly represented by *Rothia* and *Bifidobacterium*, both of which exhibit distinct patterns of abundance change along the Correa cascade. *Rothia*, a commensal genus of oral origin, shows a bidirectional pattern that is entirely dependent on *H. pylori* infection status. In *H. pylori*-positive CAG, *Rothia* abundance is typically reduced, whereas in *H. pylori*-negative CAG and IM it tends to be enriched [38,91]. This context-dependent behavior underscores the importance of *H. pylori* status as a key confounder when interpreting the abundance profiles of individual taxa. *Bifidobacterium* is increasingly recognized as a potentially protective probiotic taxon in the gastric niche because of its well-established immunomodulatory and mucosal-protective functions, despite being primarily derived from the intestine. Its relative abundance declines progressively along the Correa cascade, decreasing from 8 to 12% in the healthy stomach to 4–6% at the CAG stage and further to only 1–2% in GC [92,98]. The progressive depletion of *Bifidobacterium* has been associated with a reduction in the immunoregulatory and anti-inflammatory functions attributed to this genus, which may contribute to a shift toward a more proinflammatory gastric microenvironment [99]. Whether this relationship is causal or reflects a parallel response to shared upstream drivers, such as mucosal atrophy and intragastric pH elevation, requires further experimental investigation.

Bacteroidetes are chiefly represented by *Prevotella* and *Bacteroides*, both of which contribute to gastric mucosal homeostasis through the production of metabolites such as lactate and SCFAs [42,109]. The abundance trajectory of *Prevotella* across the Correa cascade is among the most inconsistently reported findings in gastric microbiome research. In a meta-analysis of 825 samples from six independent studies, Liu et al. reported a progressive increase in intragastric *Prevotella* abundance, from 3 to 5% in the healthy stomach to approximately 15–20% in GC (*p* < 0.05), and proposed a possible opportunistic role for this genus at advanced disease stages [92]. A co-occurrence pattern with *H. pylori* at the IM stage has also been described in at least one cohort, suggesting a possible synergistic association during the gastritis-to-carcinoma transition [86]. However, multiple biopsy-based studies have reported the opposite pattern, with *Prevotella* significantly depleted in CAG relative to healthy controls [7,8,87,91]. This directional discrepancy most likely reflects methodological heterogeneity—including differences in sampling site (gastric juice versus mucosal biopsy), disease stage, geographic origin, and the risk of oral microbiota contamination during endoscopic sampling—rather than a genuine biological inconsistency. As summarized in Table 2, the overall abundance trajectory of *Prevotella* across the Correa cascade cannot be reliably established from current cross-sectional data, and no firm conclusions regarding its functional role in CAG pathogenesis can be drawn at this stage. In contrast, the relative abundance of *Bacteroides* follows a more consistent opposing trajectory, declining from 10–15% in the healthy stomach to 5–8% during CAG and further to 2–3% in GC. At the phylum level, Bacteroidetes show an overall reduction of 60–80% [95]. This decline has been observed in both *H. pylori*-positive and *H. pylori*-negative GC patients, indicating that the loss of *Bacteroides* may represent a key commensal depletion event in GC that is not strictly dependent on *H. pylori* status [100]. As an obligate intestinal symbiont adapted to a stable enteric environment, *Bacteroides* is likely poorly suited to the progressively altered acidity and inflammatory conditions of the atrophic stomach, and its mucosal-protective functions may diminish accordingly [95,101]. Notably, studies using the INS-GAS mouse model have suggested a procarcinogenic role for Bacteroides in that experimental context [102], a finding that contrasts with the protective associations observed in human cohorts and highlights the importance of species-specific and host-specific differences when interpreting microbiota–disease relationships across model systems.

Proteobacteria are mainly represented by *Helicobacter* and *Escherichia*. As the Correa cascade progresses, declining gastric acid secretion, impairment of mucosal barrier integrity, and immune dysregulation collectively remodel the gastric ecological niche, creating conditions associated with Proteobacteria enrichment [90,110]. Enrichment of Proteobacteria-associated pathobionts, particularly *H. pylori* and *E. coli*, has been associated with mucosal immune activation and oxidative stress through the release of bacterial virulence factors and endotoxins such as urease-derived ammonia and lipopolysaccharide (LPS), which may contribute to the perpetuation of chronic gastric inflammation [94,95,111]. The relative abundance of *H. pylori* follows a characteristic nonlinear pattern along the Correa cascade, rising from a detection rate of 0–50% in asymptomatic healthy individuals to 70–90% at the CAG stage (peak detection), then declining to 40–50% at the IM stage and further to only 10–20% in GC [103,104]. This nonlinear pattern is thought to reflect progressive remodeling of the gastric niche as atrophy advances, which gradually renders the environment less favorable for *H. pylori* persistence, consistent with a “hit-and-run” model of carcinogenic initiation [103]. Strains carrying the CagA virulence factor are associated with a four- to sixfold increase in carcinogenic risk [105]. Even when eradication is performed at the IM or dysplasia stage, *H. pylori* eradication may still reduce GC risk by approximately 55% [105]. In contrast, *E. coli* accounts for only 0.5–1% of the intragastric microbial community in the healthy stomach, but increases to 3–5% at the CAG stage and rises further to 8–12% in GC(*p* < 0.001) [99,108]. The rise in intragastric pH from approximately 1.5–2.0 to 4.0–5.0 in the setting of hypochlorhydria has been proposed as a key permissive factor for retrograde translocation of intestinal bacteria, including *E. coli*, into the gastric lumen [106,107]; this inference is supported by in vitro acid-survival data and observational human studies, though formal mechanistic confirmation in human gastric tissue remains limited.

Within Fusobacteria, *Fusobacterium nucleatum* has been identified as an oral-derived opportunistic pathobiont associated with gastric carcinogenesis. Its enrichment is predominantly observed at the GC stage, with limited evidence of significant accumulation at earlier stages of the Correa cascade [88,90]. Enrichment relative to healthy controls has been confirmed in at least three independent cohorts, including a meta-analysis [90,92,95], though quantitative abundance data vary considerably across geographic cohorts and sequencing platforms, and gastric-specific mechanistic evidence remains limited.

**Figure 1:**
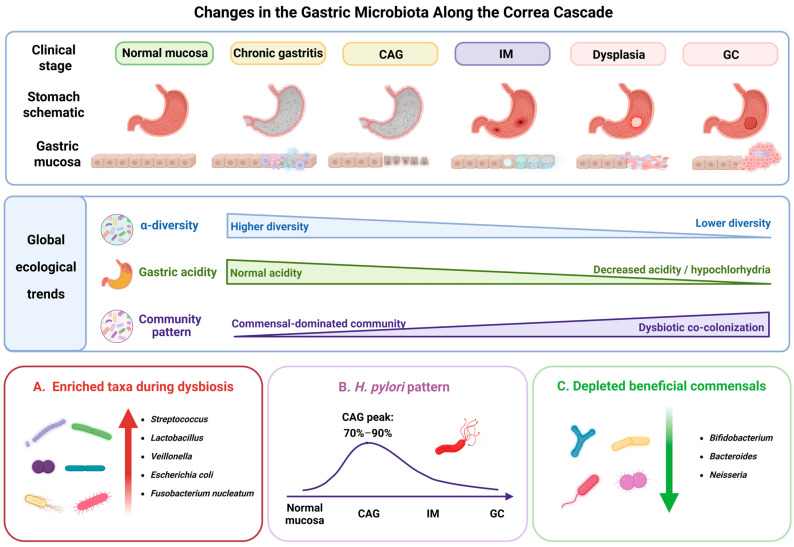
Changes in the Gastric Microbiota along the Correa cascade. Schematic overview of compositional shifts in major gastric bacterial taxa across the clinical continuum from normal mucosa through chronic gastritis, chronic atrophic gastritis (CAG), intestinal metaplasia (IM), dysplasia, and gastric cancer (GC), including representative relative-abundance ranges for *Helicobacter pylori* and qualitative directional trends for *Streptococcus*, *Lactobacillus*, *Veillonella*, *Escherichia coli*, *Fusobacterium nucleatum*, *Bacteroides*, *Bifidobacterium*, and *Neisseria*, alongside global trends in α-diversity, gastric acidity, and community pattern. Values represent approximate relative-abundance ranges or qualitative directional trends summarized from the studies cited in the main text (see Table 1 and Table 2); unreported stage-specific values were intentionally omitted. Prevotella and Rothia are not depicted with a single directional trend in this figure, as their abundance trajectories are inconsistently reported across studies (see Table 1 and Section Changes in Gastric Microbiota Composition along the Correa Cascade for details). Created in BioRender. https://BioRender.com/37pjwzg (accessed on 28 June 2026).

Taken together, available cross-sectional evidence indicates that as the gastric mucosa progresses from a healthy state through CAG and IM to GC, the intragastric microbiota undergoes substantial restructuring, transitioning from a relatively simple community in which *H. pylori* is the principal pathobiont to a more complex ecosystem characterized by co-colonization of oral- and intestinal-derived taxa. Several oral-associated genera within Firmicutes, including *Streptococcus*, *Lactobacillus*, and *Veillonella*, become progressively enriched, whereas protective commensals such as *Bifidobacterium* and *Bacteroides* decline substantially. Importantly, the directional changes reported for individual taxa—particularly *Prevotella* and *Rothia*—remain contested across studies, reflecting methodological heterogeneity and the confounding influence of *H. pylori* status, sampling approach, and geographic origin. The cross-study reproducibility of these findings is documented in Table 1, and stage-resolved quantitative trends along the Correa cascade are presented in Table 2. The compositional shifts are schematically illustrated in Figure 1.

#### 4.3.2. Mechanistic Roles of Gastric Microbiota Dysbiosis in the Pathogenesis of CAG

##### Metabolic Remodeling: Disruption of SCFA and Bile Acid Homeostasis

Metabolic dysfunction driven by gastric microbiota dysbiosis represents an important mechanistic component in the pathogenesis of CAG. In 2017, Parsons et al. used 16S rRNA sequencing combined with predictive functional profiling and found upregulation of pathways related to glucose-6-phosphate dehydrogenase and D-lactate dehydrogenase in the gastric microbiota of patients with *H. pylori*-associated AG [44]. These metabolic alterations have previously been linked to poor prognosis in GC. The same study further showed increased expression of fumarate reductase together with reduced succinate dehydrogenase activity in patients with both autoimmune and *H. pylori*-associated AG, suggesting disruption of the tricarboxylic acid (TCA) cycle [44]. In a 2025 study, Otani et al. performed metabolomic profiling and found that patients with AAG exhibited suppressed glycolytic and TCA cycle activity, accompanied by activation of alternative metabolic pathways [112]. These findings indicate substantial reprogramming of energy metabolism and suggest that such alterations are closely associated with disease progression and increased risk of malignant transformation.

Aberrant SCFA metabolism represents a key feature of microbiota-related functional disruption in CAG. SCFAs, mainly acetate, propionate, and butyrate, are generated by anaerobic bacterial fermentation of dietary fiber in the gut and play essential roles in maintaining epithelial barrier integrity, regulating energy metabolism, and limiting inflammatory responses [113]. It should be noted that the mechanistic evidence for SCFA-mediated mucosal protection cited in this section derives primarily from intestinal epithelial cell models and animal models of intestinal inflammation; while emerging evidence from murine gastric tissue models suggests that SCFAs can also act directly on gastric epithelial cells, direct validation in human gastric tissue remains limited. The discussion below therefore represents a framework of biological plausibility that requires gastric-specific human validation. The protective effects of SCFAs are mediated through several distinct mechanisms. Butyrate enhances the expression of tight-junction proteins, including claudin-1, zonula occludens-1 (ZO-1), and occludin, thereby helping to preserve epithelial barrier function. Butyrate also upregulates mucin 2 expression and promotes the thickening of the mucus layer. Beyond barrier protection, SCFAs regulate gene expression by inhibiting histone deacetylases (HDACs) and exert immunoregulatory effects via activation of G protein-coupled receptors, particularly GPR41/FFAR3 and GPR43/FFAR2. In CAG, depletion of SCFA-producing commensal bacteria may lead to a deficiency of these protective metabolites, which has been hypothesized to aggravate mucosal injury and perpetuate inflammatory cascades, though direct evidence in human gastric tissue remains limited. Clinical evidence further indicates that fecal SCFA levels and the composition of SCFA-producing bacterial communities undergo significant alterations in patients with gastric lesions after *H. pylori* eradication [114].

Bile acid metabolic dysregulation represents another prominent consequence of microbiota-related functional imbalance. Intestinal bacteria convert primary bile acids synthesized in the liver into secondary bile acids. Together, these bile acid species not only facilitate lipid digestion but also regulate inflammation, metabolic homeostasis, and epithelial integrity through receptors such as farnesoid X receptor (FXR) and Takeda G protein-coupled receptor 5 (TGR5) [115]. When gastrointestinal microbial homeostasis is disrupted or when bile reflux exposes the gastric mucosa to excessive bile acids, aberrant bile acid accumulation may directly damage the mucosa, induce DNA injury and oxidative stress, and reshape barriers and immune functions in ways that favor the progression along the atrophy–intestinal metaplasia–carcinoma sequence [116]. In the setting of CAG, such bile acid perturbations may further aggravate mucosal injury and contribute to the establishment of a protumorigenic microenvironment through interconnected metabolic and signaling pathways. Taken together, reduced SCFA availability and disturbed bile acid metabolism may constitute a critical mechanistic link between microecological dysbiosis and pathological progression of the gastric mucosa in CAG.

##### Chronic Activation of Inflammatory Signaling Pathways

Gastric microecological dysbiosis may sustain activation of inflammatory signaling pathways through multiple converging mechanisms, thereby forming a pivotal molecular link between microbial perturbation and chronic mucosal inflammation. *H. pylori*, together with opportunistic pathogens enriched under dysbiotic conditions (e.g., *Streptococcus* spp.), engages host innate immune receptors through virulence factors and PAMPs [7,20]. Concurrently, depletion of commensal taxa, including *Lactobacillus* spp., weakens tonic suppression of the NF-κB pathway. In this regard, *L. plantarum* suppresses NF-κB activation by blocking IκBα degradation and inhibiting p65 phosphorylation [117], whereas the concomitant reduction in SCFA production may further diminish GPR43-mediated restraint of NF-κB signaling through HDAC-related mechanisms [118]. In addition, dysbiosis-associated impairment of the mucosal barrier permits greater translocation of microbial components across the epithelium, thereby amplifying innate immune activation in the lamina propria [119].

Driven by microbiota dysbiosis, the NF-κB signaling pathway serves as a central hub linking gastric microecological imbalance to chronic inflammation. This pathway undergoes sustained activation and plays a critical role in *H. pylori*-associated gastric pathology and gastrointestinal tumorigenesis [120]. *H. pylori* infection activates host cell surface pattern recognition receptors (PRRs), including TLRs and NOD1, through virulence-associated components such as CagA, peptidoglycan, and VacA. This process triggers the IKK complex, leading to phosphorylation and proteasomal degradation of IκB, which in turn releases NF-κB for nuclear translocation and transcriptional activation of proinflammatory gene programs [121,122]. Importantly, non-*H. pylori* pathobionts enriched during the dysbiotic phase of CAG, such as *Streptococcus anginosus*, can also activate proinflammatory signaling pathways, including MAPK signaling, through direct interaction between bacterial surface proteins and receptors on gastric epithelial cells [20]. PAMPs produced by these bacteria, particularly LPS, may further amplify NF-κB activation via the TLR4 axis [123], making NF-κB signaling under dysbiotic conditions both multifactorial and persistent. Once activated, NF-κB transcriptionally upregulates a broad range of proinflammatory mediators, including cytokines (IL-8, TNF-α, and IL-1β), chemokines (CXCL1 and CXCL2), and adhesion molecules (ICAM-1 and VCAM-1). These factors collectively recruit neutrophils and monocytes into the gastric mucosa, thereby aggravating inflammatory tissue injury [124].

In patients with CAG and long-standing *H. pylori* infection, NF-κB activity is markedly increased and positively correlated with neutrophil infiltration, inflammatory severity, and the degree of glandular atrophy in the gastric mucosa, underscoring the central role of this dysbiosis-related pathway in maintaining a chronic inflammatory microenvironment and promoting pathological progression [120]. Consistent with these findings, gastric microbiota diversity is substantially reduced in CAG, whereas *Streptococcus* abundance is increased and positively associated with more advanced OLGA/OLGIM staging [7], suggesting that the enrichment of opportunistic pathobionts may contribute to NF-κB-driven inflammatory progression. Importantly, NF-κB activation induced by microbiota dysbiosis extends beyond acute and chronic inflammation and immune activation. It also contributes to genomic instability by promoting R-loop formation and DNA double-strand breaks, thereby providing a molecular basis for inflammation-associated cellular transformation and carcinogenesis [125]. Collectively, microecological imbalance perpetuates a self-reinforcing cycle of “inflammation, injury, and aberrant repair” through recurrent or sustained NF-κB activation, thereby serving as a critical mechanistic bridge in the progression from CAG to IM and more advanced neoplastic stages.

Persistent activation of inflammatory signaling pathways driven by microbiota dysbiosis ultimately leads to the sustained release of proinflammatory cytokines, thereby providing a biological basis for chronic mucosal inflammation. Within the dysbiotic microenvironment, sustained colonization by pathogenic microorganisms, together with the loss of protective functions normally provided by commensal bacteria, has been associated with sustained elevation of proinflammatory mediators, such as TGF-β, TNF-α, and IL-6, each of which has been implicated in the development and progression of CAG in human observational and experimental studies [22]. At the molecular level, sustained immune activation triggered by microbiota disruption drives several key cytokines to promote atrophy and intestinal metaplasia through distinct pathways. Interferon-γ (IFN-γ) directly induces gastric epithelial cell death and accelerates the metaplastic process [126]. In contrast, IL-1β promotes glandular atrophy by suppressing parietal cell-associated Sonic Hedgehog signaling and reducing proton pump activity [127]. In addition, enhanced Th17 cell polarization under dysbiotic conditions has been reported in both human gastric tissue studies and murine *H. pylori* infection models [128], and elevated IL-17A has been associated with parietal cell apoptosis, atrophic lesions, and metaplastic changes, primarily based on in vitro and animal model evidence [129]. *H. pylori*-derived extracellular vesicles and outer membrane vesicles (EVs/OMVs), enriched in virulence factors such as CagA and VacA, have been shown to be internalized by epithelial and immune cells, stimulating the production of proinflammatory cytokines including TNF-α, IL-6, IL-1β, and IL-8, as demonstrated primarily using in vitro co-culture systems [130]. Environmental and dietary factors may further modulate this cytokine cascade. For example, a high-salt diet has been shown to markedly potentiate the inflammatory response induced by *H. pylori* infection, increasing the expression of TNF-α, IL-1β, IL-6, and IFN-γ, aggravating mucosal injury, and accelerating pathological progression [131].

##### Immune Imbalance and Oxidative Stress

Microbiota dysbiosis represents an important upstream driver of immune imbalance and oxidative stress in CAG. As discussed above, the gastric microbiota in CAG is characterized by the enrichment of pathogenic bacteria and depletion of commensal organisms. This dysbiosis disrupts the immune system and redox homeostasis through multiple interconnected pathways. On one hand, pathogenic bacteria and their products, including LPS and peptidoglycan, continuously activate innate immunity via PRRs, thereby disrupting the Th17/Treg balance and sustaining chronic inflammation [132]. In contrast, the loss of commensal bacteria leads to a deficiency in immunomodulatory metabolites, particularly SCFAs, which impairs the differentiation and functional maintenance of regulatory T cells (Tregs). Furthermore, sustained immune activation induced by dysbiosis markedly increases the generation of ROS and activates the NLRP3 inflammasome, establishing a vicious cycle of “microbiota dysbiosis, immune imbalance, oxidative stress, tissue injury, and further microbiota dysbiosis.” The following sections discuss this process from three dimensions: imbalance of the Th17/Treg axis, metabolic reprogramming of immune cells, and oxidative stress-mediated activation of the NLRP3 inflammasome.

Th17/Treg Axis Dysregulation

Gastric microbiota dysbiosis has been associated with Th17/Treg axis imbalance. Under homeostatic conditions, commensal bacteria have been shown to promote the differentiation of naïve CD4^+^ T cells into Foxp3^+^ Tregs through the production of SCFAs, particularly butyrate, which activates GPR43 and inhibits histone deacetylase (HDAC) activity [133]. This mechanistic pathway is well established in murine intestinal models and human immune cell systems, including studies using human dendritic cell–T cell co-culture; however, direct evidence from human gastric tissue is currently lacking, and whether this mechanism operates within the gastric mucosal microenvironment at physiologically relevant SCFA concentrations remains to be determined. In this context, depletion of butyrate-producing bacteria, such as *Faecalibacterium* and *Roseburia*, which primarily inhabit the intestinal tract but have been reported to be altered in the gastrointestinal microenvironment of CAG patients, has been associated with reduced butyrate availability, which may impair Treg induction. Direct evidence that these genera are specifically depleted within the gastric mucosal niche itself, rather than within the broader intestinal compartment of CAG patients, has not been established. Concurrently, *H. pylori* and other enriched pathogenic taxa, including *Streptococcus* and *Prevotella*, activate dendritic cells through PAMPs, promoting the secretion of Th17-polarizing cytokines, such as IL-6 and IL-23, which, in turn, drive excessive differentiation of Th17 cells and increased release of IL-17 [134]. This dysbiosis-mediated dual imbalance, characterized by insufficient Treg induction and Th17 activation, represents a central feature of immune pathology in patients with CAG.

The imbalance of the Th17/Treg axis constitutes a central mechanism in the immunoregulation of CAG. In a seminal 2008 study, Stummvoll et al. were the first to systematically delineate the distinct roles of different T-cell subsets in autoimmune gastritis and their differential susceptibility to suppression by Tregs [135]. Using a transgenic TCR mouse model (TxA23), the authors transferred fully differentiated Th1, Th2, and Th17 cells recognizing the parietal cell antigen H^+^/K^+^-ATPase into immunodeficient recipients and demonstrated that all three effector lineages were capable of inducing autoimmune gastritis, albeit with markedly distinct pathological patterns. Among them, Th17 cells induced the most destructive disease, characterized by eosinophil-predominant cellular infiltration and markedly elevated serum IgE levels. More importantly, polyclonal Tregs showed markedly different suppressive capacities toward the three effector lineages: they effectively inhibited Th1-mediated disease, moderately suppressed Th2-driven pathology, but were able to restrain Th17-mediated disease only at early time points. These findings indicate that Th17 cells possess distinctive pathogenicity and relative resistance to immunoregulatory suppression in CAG. These observations derive from an antigen-specific murine model and await validation in human autoimmune atrophic gastritis cohorts before direct clinical extrapolation.

Subsequently, Huter et al. (2009) demonstrated in a murine adoptive transfer model that antigen-specific induced regulatory T cells (iTregs) have greater suppressive capacity than polyclonal Tregs and can effectively prevent both Th1- and Th17-mediated autoimmune gastritis, providing an experimental basis for Treg-based cellular immunotherapy; human clinical validation of this approach in autoimmune atrophic gastritis has not yet been reported [136]. In a comprehensive review published in 2024, Vavallo et al. summarized recent advances and emphasized that, in addition to Th1 cells, Th2 cells, Th17 cells, macrophages, epithelial cells, and their derived mediators and cytokines, including IL-4, IL-9, IL-12, and IL-17, may all contribute to the pathogenesis of AAG [137].

Disruption of the Th17/Treg balance is observed not only in AAG, but is also of substantial pathological relevance in *H. pylori*-associated CAG. Importantly, this imbalance is closely linked to microbiota dysbiosis: *H. pylori* infection not only directly activates pathways driving Th17 differentiation but also indirectly amplifies Th17 responses by reshaping the gastric microecological environment, for instance, by reducing microbial diversity and promoting the enrichment of proinflammatory bacteria. Concurrently, depletion of SCFAs and tryptophan-derived metabolites caused by microbiota dysbiosis further impairs the maintenance of Treg numbers and their suppressive function [138], reducing their ability to counterbalance overactivated effector T cells and ultimately establishing a vicious cycle of chronic inflammation and tissue injury. Understanding this proposed mechanistic link between microbiota dysbiosis and immune imbalance may provide important guidance for the development of immunotherapeutic strategies based on microecological modulation.

Immune Cell Metabolic Reprogramming and Functional Impairment

Microbiota dysbiosis profoundly influences the metabolic reprogramming of immune cells by altering the supply of metabolic substrates and the signaling microenvironment. The mechanistic framework described in this subsection is largely derived from studies of systemic and intestinal immunity; direct evidence for dysbiosis-driven immunometabolic reprogramming specifically within the gastric mucosal microenvironment remains limited and largely inferential at present. In the normal gastric mucosa, SCFAs produced by commensal bacteria, particularly butyrate and propionate, serve as critical fuels for mitochondrial oxidative phosphorylation and act as HDAC inhibitors, playing essential roles in the metabolic maintenance of Tregs and the polarization of M2-type macrophages [139]. The depletion of SCFA-producing bacteria in the stomachs of patients with chronic atrophic gastritis (CAG) directly results in insufficient substrates for oxidative phosphorylation (OXPHOS) and fatty acid oxidation (FAO) pathways, which Tregs depend on to maintain their suppressive function. Concurrently, LPS and other pro-inflammatory molecules generated by the overgrowth of pathogenic bacteria continuously activate macrophages and effector T cells, forcing them to sustain a highly glycolytic metabolic state and accelerating metabolic exhaustion. Furthermore, local nutrient competition (for glutamine, arginine, and tryptophan) and lactate accumulation caused by dysbiosis further disrupt the metabolic adaptation of immune cells, establishing a microbiota-driven state of immunometabolic dysfunction.

The metabolic state of immune cells closely determines their functional performance, and aberrant metabolic reprogramming represents a critical mechanism underlying immune imbalance in CAG. Immune cell activation requires a metabolic shift from an OXPHOS-dominated to a glycolysis-dominated mode, a transition coordinated by key transcription factors, including hypoxia-inducible factor 1α (HIF-1α), mTOR, and NF-κB. A comprehensive review by Morris et al. systematically elucidated how redox mechanisms regulate the metabolic reprogramming of immune cells. The authors demonstrated that ROS can function as signaling molecules to promote HIF-1α stabilization and the glycolytic switch, while simultaneously impairing the mitochondrial electron transport chain, thereby inducing metabolic imbalance. This dual role of ROS is particularly prominent in chronic inflammatory microenvironments [140].

In the inflammatory microenvironment of CAG, the metabolic reprogramming of macrophages is particularly pronounced and intimately associated with microbiota dysbiosis. Sustained stimulation by pathogen-derived PAMPs, such as LPS, drives macrophage polarization toward the proinflammatory M1 phenotype. M1-polarized macrophages predominantly rely on glycolysis and the pentose phosphate pathway (PPP) for energy production and biosynthetic precursors, whereas their tricarboxylic acid (TCA) cycle undergoes characteristic disruption, leading to the accumulation of succinate and citrate. Succinate accumulation promotes the transcription of pro-inflammatory cytokines, such as IL-1β, through the stabilization of HIF-1α, whereas citrate provides substrates for fatty acid synthesis that support the production of inflammatory mediators [141]. Conversely, anti-inflammatory M2-type macrophages depend on an intact TCA cycle and FAO to sustain their function, and SCFAs produced by commensal bacteria represent key substrates supporting the M2 metabolic phenotype. The reduction in SCFAs resulting from dysbiosis exacerbates the imbalance in M1/M2 polarization [142].

The metabolic reprogramming of T cells is subject to precise regulation. Activated effector T cells (including the Th1, Th2, and Th17 subsets) must upregulate glycolysis and glutamine metabolism to meet the demands of rapid proliferation and effector molecule synthesis, whereas Tregs primarily depend on OXPHOS and FAO to maintain their suppressive function and long-term survival. Recent studies have demonstrated that T cells in chronic inflammatory environments may undergo metabolic exhaustion, characterized by mitochondrial dysfunction, reduced glycolytic capacity, and impairment of antioxidant defense systems. This metabolic exhaustion is closely associated with immunosenescence, resulting in impaired immune cell function and reduced pathogen clearance capacity [143].

In CAG, persistent *H. pylori* infection and the consequent microbiota dysbiosis collectively drive metabolic disturbances in both T cells and macrophages. The depletion of critical amino acids, such as glutamine and arginine, restricts the proliferative capacity of effector T cells, whereas nutrient competition compromises the immunosuppressive function of Tregs. Moreover, lactate accumulation within the inflammatory microenvironment lowers local pH and inhibits the mTOR signaling pathway, further impairing the metabolic adaptability of T cells. These microbiota dysbiosis-driven metabolic impairments not only attenuate anti-infection immune responses but may also promote the survival of autoreactive T cells through disruption of central immune tolerance mechanisms, thereby exacerbating tissue damage.

Oxidative Stress and Activation of the NLRP3 Inflammasome

Microbiota dysbiosis may contribute to exacerbated oxidative stress in CAG. Under healthy conditions, antioxidant metabolites derived from commensal bacteria, such as butyrate, which activates the NRF2 pathway [144], and indole derivatives with direct ROS-scavenging capacity, cooperate with the host’s endogenous antioxidant defense system to maintain redox homeostasis. However, gastric commensal depletion in patients with CAG substantially weakens microbiota-derived antioxidant protection. Concurrently, enriched pathogenic bacteria, including *H. pylori* through its CagA and VacA virulence factors, and certain *Streptococcus* and *Escherichia–Shigella* species via LPS and other endotoxins, persistently activate the respiratory burst of neutrophils and macrophages, resulting in the massive release of ROS and RNS. This convergence of weakened antioxidant defense and intensified pro-oxidative assault drives the gastric mucosa into a state of sustained oxidative stress, which further amplifies inflammatory damage through the activation of the NLRP3 inflammasome.

Oxidative stress plays a dual role in the development and progression of atrophic gastritis, serving not only as a direct mediator of tissue injury but also as an important driver of immune dysregulation. Excessive production of ROS and reactive nitrogen species (RNS) can directly damage gastric mucosal epithelial cells, causing oxidative DNA damage, lipid peroxidation, and protein modification, thereby promoting cellular apoptosis and tissue atrophy. Chronic oxidative stress leads to intracellular ROS accumulation in T cells, accompanied by reduced levels of antioxidant enzymes, including catalase, Cu/Zn-SOD, and Mn-SOD, ultimately resulting in T cell dysfunction and apoptosis [145].

The NLRP3 inflammasome plays a pivotal role in oxidative stress-induced immune activation. The review by Bai et al. systematically explained how ROS activate the NLRP3 inflammasome [146]. This review highlighted that ROS are among the earliest intermediate products generated by many NLRP3 inflammasome activators, triggering inflammasome assembly and activation through multiple mechanisms. Specifically, ROS induce the dissociation of thioredoxin-interacting protein (TXNIP) from thioredoxin (TRX), after which TXNIP directly binds to NLRP3 to initiate inflammasome assembly. The activated NLRP3 inflammasome promotes caspase-1 activation, which in turn catalyzes the maturation and release of pro-IL-1β and pro-IL-18, triggering a robust inflammatory response.

Kodi et al. further elucidated the complex regulatory network governing NLRP3 inflammasome activation [147]. This review noted that NLRP3 inflammasome activation requires two sequential signals: the first (the priming signal) activates the NF-κB pathway via TLRs, upregulating the expression of NLRP3 and pro-IL-1β; the second (the activation signal) is provided by diverse stimuli, including potassium efflux, mitochondrial dysfunction, mitochondrial ROS (mtROS) release, and lysosomal rupture. In atrophic gastritis, *H. pylori* infection can simultaneously provide both signals through its virulence factors (such as CagA and VacA), thereby activating the NLRP3 inflammasome within the gastric mucosa. It should be noted that direct evidence for NLRP3 inflammasome activation triggered specifically by non-*H. pylori* microbiota dysbiosis in human gastric tissue remains limited; the mechanistic framework described above is primarily informed by in vitro inflammasome assembly assays and *H. pylori*-specific experimental models.

Notably, a positive feedback loop exists between oxidative stress and NLRP3 inflammasome activation. ROS directly activate the NLRP3 inflammasome, while the subsequent release of IL-1β and IL-18 stimulates immune cells and epithelial cells to generate additional ROS, thereby perpetuating a vicious cycle of oxidative stress and inflammation. A review by Liu et al. underscored that mitochondrial dysfunction constitutes the pivotal nexus of this vicious cycle [148]. Oxidative damage to mitochondrial DNA (mtDNA) and the mitochondrial permeability transition lead to the massive release of mtROS and leakage of mtDNA into the cytosol; the latter activates type I interferon responses through the cGAS-STING pathway, further amplifying the inflammatory cascade.

Regarding antioxidant defense in atrophic gastritis, the nuclear factor erythroid 2-related factor 2 (Nrf2) system serves as a critical protective mechanism. Nrf2 is the master transcription factor governing cellular antioxidant responses by upregulating the expression of multiple antioxidant enzymes, including glutamate-cysteine ligase, superoxide dismutase, catalase, and heme oxygenase-1 (HO-1). However, chronic oxidative stress and sustained inflammatory stimulation can impair the function of the Nrf2 system or induce compensatory dysregulation, thereby compromising host ROS-scavenging capacity. Furthermore, an antagonistic relationship exists between Nrf2 activation and NLRP3 inflammasome activity, in which Nrf2 activation suppresses NLRP3 inflammasome assembly, whereas excessive NLRP3 inflammasome activation may compromise Nrf2 function [149], suggesting that the balance between these two pathways is essential for maintaining gastric mucosal homeostasis. From a microecological therapeutic perspective, probiotic-mediated restoration of commensal communities, which augments the supply of SCFAs and indole metabolites, has the potential to simultaneously reinforce the Nrf2 antioxidant pathway and restrain excessive NLRP3 inflammasome activation, offering a new therapeutic strategy for interrupting the vicious cycle of dysbiosis, oxidative stress, and inflammation in atrophic gastritis.

**Figure 2:**
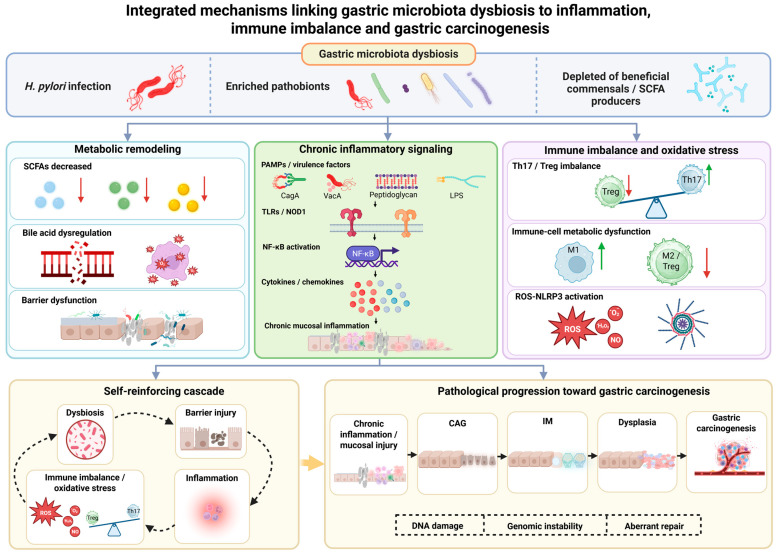
Integrated mechanisms linking gastric microbiota dysbiosis to inflammation, immune imbalance and gastric carcinogenesis. *H. pylori* infection, enrichment of opportunistic/pathobiont taxa (e.g., *Streptococcus* spp., *Escherichia coli*, *Fusobacterium nucleatum*), and depletion of beneficial commensals/SCFA producers collectively drive three converging mechanistic axes: metabolic remodeling (SCFA depletion, bile acid dysregulation, barrier dysfunction), chronic inflammatory signaling (PAMP/virulence factor-driven PRR activation, NF-κB pathway activation, and downstream cytokine and chemokine upregulation), and immune imbalance with oxidative stress (Th17/Treg imbalance, immune-cell metabolic dysfunction, and ROS-driven NLRP3 inflammasome activation). These pathways converge to sustain a self-reinforcing cascade of dysbiosis, barrier damage, chronic inflammation, and immune/oxidative dysregulation, contributing to progression along the CAG–IM–dysplasia continuum toward gastric carcinogenesis, with cumulative DNA damage, genomic instability, and aberrant tissue repair representing key molecular substrates underlying this transition. Created in BioRender. https://BioRender.com/g0r9krk (accessed on 28 June 2026).

In summary, gastric microbiota dysbiosis may participate in the onset and progression of CAG through the synergistic interplay of metabolic functional remodeling, sustained inflammatory signaling activation, and the convergence of immune dysregulation with oxidative stress. The depletion of SCFA-producing beneficial bacteria, together with aberrant bile acid metabolism, weakens mucosal barrier protection and immunoregulatory capacity. Concurrently, pathogen enrichment and commensal depletion result in multifactorial and persistent activation of inflammatory pathways, such as NF-κB, driving sustained proinflammatory cytokine release. In parallel, dysbiosis disrupts the Th17/Treg balance, causes metabolic reprogramming defects in immune cells, and establishes a positive feedback loop through excessive ROS production and NLRP3 inflammasome activation. These three mechanisms are intimately interconnected and progressively amplify one another, collectively establishing a self-reinforcing pathological cascade spanning microbiota dysbiosis, metabolic derangement, chronic inflammation, immune dysregulation, oxidative damage, and progression toward gastric carcinogenesis. This integrated framework advances our understanding of CAG pathogenesis from a microecological perspective and provides a conceptual basis for the development of targeted microbiota-directed interventions. These integrated mechanisms are schematically illustrated in Figure 2.

#### 4.3.3. The Gastric-Gut Axis and Inter-Organ Microecological Dysregulation

The mechanisms discussed above primarily focus on the pathogenic role of local gastric microecological dysbiosis. However, an accumulating body of evidence indicates the existence of a bidirectional microecological regulatory network between the stomach and the intestine, termed the “gastric-gut axis,” whose functional disruption may constitute an integral component of microecological disturbance in CAG [150]. Dysbiosis of the intestinal microbiota may influence the progression of gastric lesions through microbial metabolite signaling and immunomodulatory pathways.

##### Influence of the Stomach on the Intestinal Microbiota

Under normal physiological conditions, gastric acid (pH 1.5–3.5) serves as the first microbial barrier of the digestive tract, functioning as an “ecological filter” that efficiently eliminates exogenous microorganisms ingested orally and thereby maintains the structural stability of downstream intestinal communities [151]. In patients with CAG, however, progressive loss of parietal cells leads to hypochlorhydria, substantially compromising this ecological filtering capacity. In a preliminary cross-sectional study, Filardo et al. demonstrated a significant positive correlation between intragastric pH and duodenal microbiota α-diversity across patients with varying degrees of gastric mucosal alteration; hypochlorhydria, which was most prevalent in CAG patients, was most strongly associated with increased duodenal microbial diversity [152].The hypoacidic environment was further associated with the presence of oral-derived bacteria in the duodenal microbiota, including *Rothia mucilaginosa*, *Streptococcus salivarius*, and *Granulicatella adiacens*, suggesting that a low-acid gastric milieu may act as a contributive factor for duodenal dysbiosis. These findings require replication in larger prospective cohorts before definitive conclusions can be drawn. One clinical study further reported a small intestinal bacterial overgrowth (SIBO) detection rate of 57.5% among patients with CAG and hypochlorhydria [153], causing symptoms including abdominal distension, diarrhea, and malabsorption that collectively exacerbate the overall disease burden; this finding requires confirmation in independent cohorts given the considerable methodological variability in SIBO diagnostic criteria across studies. Beyond the acid-dependent pathway, *H. pylori* infection exerts systemic effects on the distal intestinal microbiota through alterations in gastric acidity and the local immune microenvironment. Multiple studies have demonstrated that the fecal microbiota of *H. pylori*-infected individuals differs significantly from that of uninfected controls, manifesting as compositional shifts and an overall increase in diversity [154,155]. Moreover, systemic antibiotic regimens employed for *H. pylori* eradication can transiently alter the composition of the intestinal microbiota, typically manifesting as an expansion of *Proteobacteria* and a concomitant reduction in *Actinobacteria* in the short term (within 1–3 months following treatment completion). Evidence from multiple eradication studies and their meta-analyses suggests that these perturbations largely resolve within 6 months to 1 year, with no significant phylum-level differences detectable at ≥1-year follow-up [156].

##### Influence of the Intestinal Microbiota on the Stomach

The evidence presented above demonstrates that alterations in the gastric environment can profoundly reshape downstream intestinal microbiota composition. Notably, this influence is bidirectional. Metabolites derived from intestinal microbiota can, in turn, act on the gastric mucosa through multiple pathways, thereby constituting a bidirectional regulatory network of gastrointestinal microecology. The most representative of these pathways involves bile acid–microbiota interactions. Primary bile acids synthesized by the liver undergo biotransformation by intestinal bacteria into secondary bile acids, including deoxycholic acid and lithocholic acid, which can reflux into the gastric lumen under conditions of duodenogastric reflux (DGR). Huang et al. found that bile reflux significantly increased gastric mucosal microbial diversity and altered community structure in patients with atrophic gastritis, an effect substantially more pronounced than that observed in patients with non-atrophic gastritis. This disparity is attributable to a synergistic elevation of intragastric pH resulting from the convergence of atrophy-induced hypochlorhydria and the alkaline properties of refluxed bile [157]. Wang et al. conducted an integrated study combining clinical gastric juice analysis, in vitro GES-1 cell assays, and murine models, demonstrating that taurodeoxycholic acid (TDCA), enriched in the gastric juice of bile reflux gastritis and GC patients, was significantly and positively correlated with the abundance of LPS-producing bacteria such as *Prevotella melaninogenica*. In vitro, TDCA promoted GES-1 proliferation through activation of the IL-6/JAK1/STAT3 pro-inflammatory signaling axis, and promotion of gastric precancerous progression was further corroborated in murine gavage and surgical bile reflux models [158]. These findings are consistent with the hypothesis that secondary bile acids derived from intestinal microbiota may contribute to gastric precancerous progression via the reflux route, though direct causal demonstration in human CAG patients remains limited to correlational evidence. In addition, intestinal dysbiosis has been associated with increased systemic translocation of LPS and proinflammatory cytokines, including IL-1β, IL-6, and TNF-α, as demonstrated primarily in animal models of intestinal permeability and human observational studies of metabolic endotoxemia. These circulating mediators have been proposed to act upon the gastric mucosa via the systemic circulation, potentially activating inflammatory cascades including the NF-κB pathway and thereby synergizing with the local mechanisms described in Section Chronic Activation of Inflammatory Signaling Pathways [24]; however, direct evidence specifically linking intestinal dysbiosis-derived systemic mediators to gastric mucosal NF-κB activation in human CAG patients has not been established. SCFAs produced through fermentation of dietary fiber by intestinal bacteria exert well-documented anti-inflammatory and mucosal barrier-protective effects. Dysbiosis-associated reductions in SCFA production may therefore attenuate systemic anti-inflammatory tone and indirectly aggravate gastric mucosal injury [159].

In summary, current observational and experimental evidence is consistent with the existence of a cross-organ bidirectional dysregulation in CAG: loss of the gastric acid barrier has been associated with intestinal dysbiosis, which may in turn generate aberrant metabolites that reach the stomach via bile reflux and systemic circulation, potentially aggravating gastric mucosal damage. It should be noted that direct causal evidence for this proposed feedback dynamic in human CAG patients remains limited, with most mechanistic support derived from animal models and cross-sectional observational studies. This conceptual framework underscores that microecological intervention in CAG should not be confined to the gastric compartment alone. Rather, a holistic perspective encompassing the gastric-gut axis is warranted, with concurrent attention directed toward the coordinated restoration of both gastric and intestinal microbial communities.

## 5. Microecological Therapeutics for CAG: From Mechanistic Insights to Clinical Applications

### 5.1. Therapeutic Limitations in CAG and the Clinical Rationale for Microecological Intervention

#### 5.1.1. Conventional Therapeutic Approaches for CAG and Their Limitations

The clinical management of CAG remains centered on etiological treatment and symptomatic supportive care [2]. For CAG associated with *H. pylori* infection, antibiotic eradication is the recommended first-line strategy [2]. Currently, the most commonly used *H. pylori* eradication regimens include bismuth-containing quadruple therapy (a PPI, a bismuth salt, and two antibiotics) and standard triple therapy (a PPI and two antibiotics) [160]. In traditional Chinese medicine (TCM) practice, spleen-strengthening and qi-replenishing formulations, such as Huangqi Jianzhong Tang and Sijunzi Tang, have demonstrated some efficacy in improving clinical symptoms and gastric mucosal pathological changes in patients with CAG [161,162,163].

Conventional therapies have important limitations. The growing prevalence of antibiotic resistance poses a major challenge to *H. pylori* eradication, as resistance rates to agents such as clarithromycin and levofloxacin continue to rise, leading to declining eradication success [164]. In addition, triple or quadruple regimens inevitably cause intestinal microecological disturbance. In antibiotic-treated CAG rat models, the abundance and diversity of gastric microbiota were markedly reduced; notably, Firmicutes, the predominant phylum prior to treatment, decreased from 85.1% to 21.9% [1]. Eradication therapy is frequently accompanied by gastrointestinal adverse effects, with approximately 20–30% of patients experiencing at least one symptom, such as diarrhea, nausea, or abdominal pain, thereby compromising quality of life and treatment adherence [165,166,167]. Probiotic supplementation has been shown to significantly reduce the incidence of these antibiotic-associated adverse effects, including diarrhea and nausea, in multiple RCTs and meta-analyses [168,169]. Beyond antibiotic-induced disturbance, long-term PPI use independently alters the composition of the gastric and gut microbiota, characterized by reduced microbial diversity and a relative expansion of oral- and intestinal-derived taxa [83,170]. Such microbiota alterations have been observationally associated with an elevated risk of GC in multiple epidemiological studies and meta-analyses [171,172,173]; however, all available evidence derives from observational designs and is susceptible to confounding by indication and reverse causation, as patients prescribed long-term PPIs frequently harbor pre-existing gastric pathology. Whether PPI-induced dysbiosis constitutes an independent mechanistic contributor to gastric carcinogenesis, or primarily reflects the confounding influence of the underlying disease burden, remains unresolved and warrants prospective investigation. Evidence from in vitro co-culture experiments [174], animal models [175,176], and human randomized controlled trials [177] collectively indicates that selected probiotic strains can suppress *H. pylori* colonization and attenuate gastric mucosal inflammation through competitive exclusion, production of antimicrobial substances, and modulation of host immune responses [178,179]. The strength of this evidence varies across study types, and findings from cell or animal models cannot be uncritically extrapolated to the human gastric environment. Consistent with these mechanistic findings, recent randomized controlled trials (RCTs) and meta-analyses have indicated that probiotic co-administration as an adjunct to *H. pylori* eradication regimens significantly improves eradication rates while reducing antibiotic-associated adverse effects [168,169].

In summary, the strongest current evidence supports probiotic supplementation as an adjunct to *H. pylori* eradication regimens, where it modestly improves eradication rates and reduces antibiotic-associated adverse effects [168,169]. Evidence that microecological interventions can independently reverse established gastric atrophy or intestinal metaplasia, or meaningfully reduce long-term gastric cancer risk, remains scarce and methodologically limited. These evidence gaps highlight the need for rigorously designed trials incorporating histological endpoints before microecological strategies can be formally integrated into CAG management guidelines.

#### 5.1.2. Current Clinical Evidence and Advances in Microecological Interventions for CAG

Published studies investigating probiotic or microbiota-based interventions in CAG or *H. pylori*-associated models predominantly involve *Lactobacillus* and *Bifidobacterium* strains, evaluated across in vitro, animal, and human study designs. Intervention modalities include in vitro coculture, oral gavage in animal models, and oral capsule administration in human trials, with the oral route being the most commonly employed. Considerable heterogeneity exists among studies with respect to strain selection, dosage, dosing frequency, intervention duration, and follow-up period, and a standardized protocol remains lacking. For human studies, the primary endpoint is explicitly specified to distinguish histological outcomes, such as atrophy or intestinal metaplasia regression, from symptomatic endpoints, since symptom improvement alone does not indicate reversal of mucosal atrophy or metaplasia. These representative experimental and clinical studies are summarized in Table 3.

### 5.2. Potential Applications of Microecological Agents in CAG

In recent years, the concept of microecological agents has expanded beyond traditional live bacterial preparations (probiotics) to encompass a broader product portfolio that includes prebiotics, synbiotics, and postbiotics [167,181]. Probiotics are live microorganisms that, when administered in adequate amounts, confer health benefits on the host [182]. The genera most widely studied in the gastric context include *Lactobacillus*, *Bifidobacterium*, selected species of *Streptococcus*, and the yeast *Saccharomyces boulardii*. In contrast, prebiotics are non-digestible dietary substrates that selectively stimulate the growth and metabolic activity of beneficial commensals, particularly *Lactobacillus* and *Bifidobacterium* species. Representative compounds include inulin, fructo-oligosaccharides (FOS), galacto-oligosaccharides (GOS), and xylooligosaccharides (XOS), each of which has been shown to reshape gastrointestinal community structure and functional output [183,184]. Synbiotics are composite formulations that combine probiotics with prebiotics. Through the synergistic pairing of beneficial organisms with their cognate substrates, synbiotics enhance the colonization capacity and metabolic activity of both exogenous and endogenous probiotic strains within the gastrointestinal tract, offering a more effective approach to restoring host microecological structure and function [185,186,187]. Postbiotics are non-viable microbial preparations comprising inactivated microorganisms, cellular structural components, and metabolic byproducts such as SCFAs, bacteriocins, peptides, and cell wall polysaccharides. Owing to their superior stability, favorable safety profile, and relatively well-characterized mechanisms of action, postbiotics are increasingly recognized as a promising frontier in microecological intervention [185,188,189].

In the context of CAG prevention and management, the aforementioned microecological agents each demonstrate distinct potential value, though their clinical evidence bases differ considerably in maturity and CAG specificity. Among the four categories, probiotics possess the most robust clinical evidence base in the CAG context, supported by multiple RCTs and meta-analyses [168,169,190]. Probiotics colonize the gastric mucosa and intestinal tract, where they suppress opportunistic pathogens, facilitate the restoration of beneficial commensal communities, and enhance the production of functional metabolites such as SCFAs. These combined actions improve the gastric mucosal microenvironment, attenuate inflammatory responses, and serve as adjunctive therapy during *H. pylori* eradication, with probiotic supplementation shown to increase eradication rates by 10–15% and reduce antibiotic-associated adverse effects [190]. Prebiotics selectively promote the expansion of endogenous beneficial bacteria, thereby reinforcing gastrointestinal barrier function and metabolic activity while reducing dependence on exogenous live bacterial supplementation [183,184]; however, CAG-specific RCT evidence for prebiotics remains limited, and their anti-inflammatory benefits in the gastric context are considered indirect, primarily mediated through SCFA production. Synbiotics achieve synergistic enhancement through the coordinated delivery of probiotics with their corresponding substrates, simultaneously modulating community structure and stimulating the generation of functional metabolites, including SCFAs and lactic acid, which further optimize the gastric mucosal microenvironment and immune homeostasis [187]; nonetheless, current evidence for synbiotics in CAG is largely extrapolated from probiotic trials, and synbiotic-specific clinical data in CAG remain limited. Postbiotics circumvent the inherent limitations of live bacterial colonization by directly delivering bioactive molecules to the host. This property enables postbiotics to exert immunomodulatory, anti-inflammatory, and mucosal protective effects under conditions of aberrant gastric acid secretion, immunosuppression, or concurrent antibiotic therapy. In addition, postbiotics carry a lower risk of bacteremia and translocation infection, conferring a more favorable safety profile in elderly patients and those with underlying comorbidities [191,192]. Nevertheless, human CAG-specific clinical evidence for postbiotics is currently lacking, and the available evidence remains largely preclinical.

**Figure 3:**
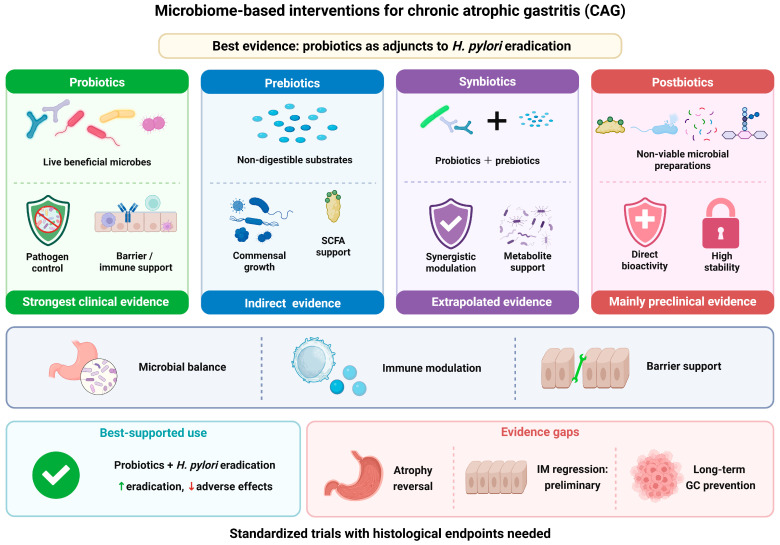
Microbiome-based interventions for chronic atrophic gastritis (CAG). Comparison of probiotics, prebiotics, synbiotics, and postbiotics with respect to representative agents, proposed mechanistic value, and current evidence in CAG. All four categories converge on three shared mechanistic axes: microecological remodeling, immunoinflammatory regulation, and mucosal barrier support/repair. Among the four categories, probiotic supplementation as an adjunct to *H. pylori* eradication has the strongest CAG-specific clinical support; evidence for direct reversal of gastric atrophy remains limited, preliminary clinical evidence for regression of intestinal metaplasia derives from a single small randomized controlled trial, and evidence for long-term gastric cancer prevention is currently insufficient. These persistent evidence gaps highlight the need for standardized trials incorporating histological endpoints to clarify the long-term disease-modifying potential of microecological interventions in CAG. Abbreviations: SCFA, short-chain fatty acid; IM, intestinal metaplasia; GC, gastric cancer. Created in BioRender. https://BioRender.com/izo3mi2 (accessed on 28 June 2026).

Accordingly, these four categories of microecological agents differ considerably in their definitions, core components, mechanisms of action, stability, safety profiles, and evidence bases; yet they also exhibit a degree of functional complementarity that may inform precision microecological intervention strategies in CAG. A systematic comparison of their characteristics, mechanisms, evidence levels, and potential clinical applications across the four categories is provided in Table 4. An overview of these four categories, their representative agents, shared mechanistic axes, and current evidence and gaps in CAG is presented in Figure 3.

### 5.3. Molecular Mechanisms Underlying Probiotic Effects in CAG

The development and progression of CAG is a complex, multifactorial, and multistage pathological process involving several core mechanisms, including gastric microecological dysbiosis, sustained chronic inflammation, and mucosal barrier damage. Probiotics, defined as live microorganisms that confer health benefits on the host when administered in adequate amounts [182], have emerged as a major focus of current research regarding their therapeutic mechanisms in CAG. Building on the clinical and experimental evidence outlined in Section 5.1, this section systematically elucidates the molecular mechanisms and functional pathways underlying probiotic interventions in CAG from three key dimensions: microecological modulation, immunoinflammatory regulation, and mucosal barrier restoration.

#### 5.3.1. Restoring Gastric Microecological Homeostasis: From Dysbiosis to Ecological Equilibrium

*H. pylori* infection is the principal initiating factor in CAG, driving gastric glandular atrophy and intestinal metaplasia through chronic inflammation and the release of virulence proteins, including CagA and VacA. Numerous studies have documented the antagonistic effects of probiotics against *H. pylori* [201]. *Lactobacillus reuteri* can directly inhibit *H. pylori* growth through the production of reuterin while competitively blocking its adhesion to gastric epithelial cells [202]. A meta-analysis by Zhou et al., encompassing eight randomized controlled trials with a total of 1087 patients, demonstrated that *L. reuteri* supplementation in standard eradication regimens significantly improved *H. pylori* eradication rates [203]. Similarly, a meta-analysis by Zhang et al. showed that probiotic supplementation combined with standard triple or quadruple therapy increased *H. pylori* eradication rates by 10–15% and significantly reduced the incidence of antibiotic-associated adverse effects [190]. Furthermore, bacteriocins and organic acids (including lactic and acetic acids) secreted by probiotics lower intragastric pH, inhibit *H. pylori* urease activity, and disrupt its survival advantage in acidic environments.

In addition to directly inhibiting pathogen proliferation, probiotics can promote the growth of other beneficial bacteria through cross-feeding mechanisms. For example, SCFAs generated by *Bifidobacterium* through dietary fiber fermentation can be utilized by other commensal bacteria such as *Butyrivibrio*, forming a mutualistic symbiotic network. As highlighted by Ouwehand et al. in their review, this “ecological niche reconstruction” effect is a key mechanism by which probiotics achieve long-term colonization and exert sustained protective effects [204].

Gastric microecological dysbiosis is manifested not only by alterations in microbial community structure but, more importantly, by disruptions in metabolic function. In patients with CAG, beneficial metabolites such as SCFAs and tryptophan metabolites are significantly reduced in the gastric lumen, whereas inflammation-associated metabolites are aberrantly elevated. Probiotic interventions can reshape the intragastric metabolite profile and restore metabolic homeostasis. First, SCFAs, as core metabolic products of probiotics, play a pivotal role in mucosal barrier repair. The SCFA family comprises acetate, propionate, and butyrate, among which butyrate exhibits the most prominent biological activity. As a primary energy source for gastrointestinal epithelial cells, butyrate promotes cellular proliferation and differentiation while enhancing mucosal barrier function. Butyrate suppresses carcinogenesis and inflammation by blocking NF-κB signaling activation and by promoting the differentiation of IL-10-producing T cells and Tregs, an effect in which its HDAC-inhibitory activity is also implicated [205]. Butyrate also functions as a broad HDAC inhibitor, suppressing tumor cell proliferation, inducing apoptosis, and inhibiting tumor progression; whether these effects operate at effective concentrations within the human gastric lumen in CAG patients remains to be directly established [205]. In vitro studies have further demonstrated that butyrate at physiologically relevant concentrations promotes gastric epithelial cell proliferation and upregulates tight junction protein expression, including Occludin, thereby supporting mucosal barrier reinforcement [205]. Additionally, tryptophan derivatives produced through probiotic metabolism, including indole-3-propionic acid and indole-3-acetic acid, activate the aryl hydrocarbon receptor (AhR) and modulate mucosal immune cell function. Polyamines generated by probiotics, such as putrescine, spermine, and spermidine, participate in the regulation of basal cellular metabolism, cell cycle control, and antioxidant defense, contributing to the reversal of the senescent phenotype in atrophic mucosa [206]. Beyond direct probiotic fermentation, dietary polyphenols and oligosaccharides may represent additional modulators of gastrointestinal SCFAs biosynthesis. Emerging evidence suggests that polyphenols and oligosaccharides may interact to influence gut microbial community structure and are associated with alterations in SCFA production (including acetate, propionate, and butyrate), potentially through multiple complementary mechanisms, such as prebiotic-like effects and selective modulation of microbial taxa, with downstream immunomodulatory consequences [207]. These polyphenol-oligosaccharide-SCFA interactions have been primarily characterized in intestinal and early-life immune contexts; whether similar dynamics contribute to SCFA-mediated mucosal protection in chronic atrophic gastritis remains to be elucidated.

#### 5.3.2. Modulating Immunoinflammatory Responses: From Proinflammatory Cascades to Immune Homeostasis

The immunopathological hallmarks of CAG include a skewed balance among helper T-cell subsets, characterized by excessive activation of Th1/Th17 lineages and concurrent functional impairment of Tregs. Probiotics can reshape T cell differentiation trajectories by engaging dendritic cells (DCs) within mesenteric lymph nodes. Kwon et al. demonstrated that *Lactobacillus sakei* WIKIM30 induces bone marrow-derived dendritic cells (BMDCs) to upregulate tolerogenic DC markers, including PD-L1 and CD103, while simultaneously stimulating secretion of IL-10 and TGF-β. These conditioned DCs promoted the polarization of naïve CD4^+^ T cells toward Foxp3^+^ Tregs, significantly elevating the Treg proportion in the mesenteric lymph nodes compared to untreated controls in a murine atopic dermatitis model [200]. The same group further reported that L. sakei WIKIM30-conditioned DCs markedly attenuated Th1, Th2, and Th17 responses while potentiating Treg-associated signaling, thereby correcting the Th17/Treg imbalance [200]; these findings, however, derive from an atopic dermatitis model, and direct validation in CAG-relevant gastric inflammation models is required before extrapolation to the gastric mucosal context.

The NLRP3 inflammasome, a multiprotein complex central to innate immune activation, remains persistently engaged during CAG pathogenesis, driving the maturation and release of IL-1β and IL-18. Li et al. revealed that *Lactobacillus rhamnosus* GR-1 (LGR-1) upregulates the autophagy-related proteins PINK1, Parkin, and LC3-II, thereby activating PINK1/Parkin-mediated mitophagy. This selective clearance of damaged mitochondria curtails ROS generation and impedes NLRP3 inflammasome assembly [199]. In an in vitro model using bovine mammary epithelial cells, LGR-1 pretreatment substantially reduced the activation of NLRP3, ASC, and caspase-1 in *Escherichia coli*-challenged cells, with a concomitant decrease in IL-1β secretion [199]. SiRNA-mediated PINK1 knockdown abolished these protective effects, confirming that PINK1/Parkin-dependent mitophagy is the principal suppressive mechanism [199]. Although this evidence derives from a non-gastric epithelial cell model, the PINK1/Parkin-NLRP3 axis is mechanistically conserved across epithelial tissues, and the documented hyperactivation of NLRP3 in CAG gastric mucosa suggests this pathway represents a plausible mechanistic target; direct evidence in gastric epithelial cells remains to be established. In addition to intact bacterial cells, probiotic-derived EVs contribute to immunomodulation. These nanoscale membrane-bound vesicles carry bioactive cargo, including proteins, lipids, and nucleic acids, and directly act on immune cells. Through TLR2-mediated signaling, probiotic EVs promote the anti-inflammatory reprogramming of macrophages and DCs [208].

#### 5.3.3. Repairing the Gastric Mucosal Barrier and Antioxidant Defense

The structural integrity of the gastric mucosal epithelial barrier depends on tight junctions, and disruption of these junctional complexes typically occurs early in CAG pathogenesis. TJs are assembled from transmembrane proteins, including occludin, claudins, and junctional adhesion molecules (JAMs), together with cytoplasmic scaffolding proteins such as ZO-1 and ZO-2. Accumulating evidence indicates that probiotics can restore gastric mucosal barrier function by modulating the expression of these key structural components. Miyauchi et al. showed that *Lactobacillus rhamnosus* OLL2838 markedly upregulated ZO-1 expression (by 4.8-fold) and myosin light-chain kinase expression (by 3.1-fold) in intestinal epithelial cells, leading to functional recovery of the intestinal barrier [209]. In a complementary line of investigation, Liu et al. identified a novel postbiotic designated HM0539, derived from *Lactobacillus rhamnosus* GG, that reinforced intestinal barrier integrity by upregulating mucin MUC2 and the tight junction protein ZO-1; this postbiotic also conferred significant protection in a dextran sulfate sodium (DSS)-induced colitis model [210]. Collectively, these findings support the concept that probiotics strengthen the gastric mucosal barrier by promoting tight junction protein expression.

The gastric mucus layer constitutes the first line of defense, shielding the underlying epithelium from hydrochloric acid and pepsin. Its principal structural components, the gel-forming mucins MUC5AC and MUC6, are secreted by gastric mucous cells. In patients with CAG, the mucus layer is characteristically attenuated and mucous cell density is reduced. Wang et al. demonstrated that p40, a secreted protein from *Lactobacillus rhamnosus* GG, activates the epidermal growth factor receptor (EGFR) and its downstream effector Akt, thereby substantially upregulating MUC2 gene expression and mucin production in LS174T cells. In vivo, p40 administration significantly augmented mucin output in the colonic epithelium of wild-type mice and produced a visibly thicker colonic mucus layer. This effect was abolished in *Egfr^wa5^* mice harboring a dominant-negative mutation in the EGFR kinase domain, establishing the dependence of p40-mediated mucosal protection on intact EGFR signaling [197]. These data indicate that probiotics can enhance mucus barrier function through EGFR-dependent stimulation of mucin biosynthesis. More recently, Chen et al. reported that *Lacticaseibacillus casei* T1 significantly attenuated the *H. pylori*-induced overexpression of MUC5AC, IL-6, IL-8, and TNF-α in GES-1 cells and ameliorated *H. pylori*-driven gastric mucosal injury [198].

Rapid turnover of the gastric mucosal epithelium is a critical prerequisite for reversing atrophic lesions. Yan et al. elucidated the molecular mechanism by which p40 stimulates epithelial cell proliferation: p40 enhances the catalytic activity of a disintegrin and metalloproteinase domain-containing protein 17 (ADAM17), triggering the ectodomain shedding of heparin-binding epidermal growth factor-like growth factor (HB-EGF) from intestinal epithelial cells. The released HB-EGF in turn transactivates EGFR in a paracrine manner. ADAM17-deficient colonic epithelial cells failed to exhibit p40-induced EGFR transactivation; however, reintroduction of wild-type ADAM17 fully rescued this response. Conversely, knockdown of HB-EGF abrogated the capacity of p40 to transactivate EGFR and Akt, preventing apoptosis and preserving tight junction integrity [211].

**Figure 4:**
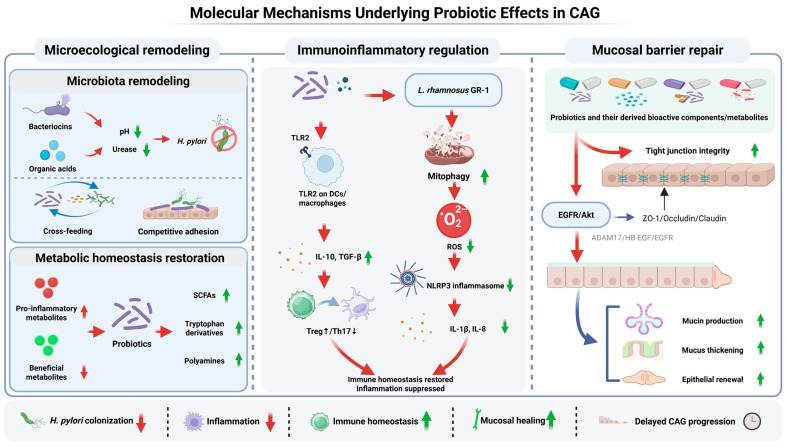
Molecular mechanisms underlying probiotic effects in chronic atrophic gastritis (CAG). Probiotics exert therapeutic effects in CAG through coordinated regulation of microecology, immunity, and mucosal barrier integrity. They remodel the gastric microbiota by inhibiting *Helicobacter pylori*, lowering pH and urease activity, and promoting beneficial microbial interactions. Metabolically, probiotics enhance the production of SCFAs, tryptophan derivatives, and polyamines while reducing pro-inflammatory metabolites. Immunologically, probiotics and their extracellular vesicles activate TLR signaling, increase anti-inflammatory cytokines (IL-10, TGF-β), restore the Treg/Th17 balance, and suppress ROS generation and NLRP3 inflammasome activation. In parallel, they strengthen epithelial barrier function via EGFR/Akt signaling, upregulate tight junction proteins, and promote mucin production and epithelial renewal. Collectively, these effects contribute to reduced inflammation, improved mucosal healing, and delayed progression of CAG. Created in BioRender. https://BioRender.com/bvtv07m (accessed on 28 June 2026).

Taken together, the evidence reviewed above suggests that probiotics may influence CAG-associated pathological processes through the coordinated engagement of three mechanistic axes: microecological remodeling, immunoinflammatory regulation, and mucosal barrier repair. At the microecological level, probiotics produce antimicrobial substances, including reuterin, bacteriocins, and organic acids, that exert antagonistic effects against *H. pylori*. Simultaneously, competitive adhesion and metabolic cross-feeding help reshape the community architecture of the gastric microbiota and restore homeostasis in metabolic circuits linked to SCFAs, tryptophan catabolites, and polyamines. At the immunological level, probiotics modulate mucosal immune tone by engaging tolerance-associated pathways, inducing a tolerogenic DC-like phenotype, and potentiating Foxp3^+^ T regulatory cell responses, thereby alleviating the Th17/Treg imbalance. Probiotics further suppress excessive NLRP3 inflammasome activation through PINK1/Parkin-mediated mitophagy, curtailing the release of pro-inflammatory mediators such as IL-1β. At the mucosal barrier level, probiotics and their derived components or metabolites (e.g., the secreted protein p40 and the postbiotic HM0539) upregulate tight-junction proteins, including ZO-1 and occludin, to preserve epithelial barrier integrity. These agents also stimulate mucin biosynthesis, particularly MUC2, via the EGFR/Akt signaling axis, thereby thickening the protective mucus layer. In parallel, transactivation of EGFR through the ADAM17/HB-EGF cascade drives epithelial proliferation and accelerates mucosal renewal. Overall, these mechanisms are more likely to operate not in isolation but as a coupled, self-reinforcing process spanning microecological reconstitution, immune homeostasis restoration, and mucosal barrier fortification. This integrated framework provides a mechanistic foundation for understanding the potential intervention value of probiotics and their metabolites in CAG and offers a theoretical basis for the development of precision microecological strategies targeting defined strains or specific bioactive components. These mechanisms are schematically illustrated in Figure 4.

### 5.4. Targeted Microecological Modulation in Integrative Chinese-Western Medicine

Microecological and systems biology tools increasingly enable systematic investigation of the multi-component, multi-target, and multi-pathway therapeutic logic of TCM in CAG. An increasing number of studies have demonstrated that classical TCM formulae reshape gastrointestinal microbial architecture and its metabolic output, thereby recalibrating host inflammatory and immune homeostatic circuits and reversing mucosal pathological damage. Zhou et al. integrated metabolomic profiling with 16S rRNA gene sequencing and showed that the Huazhuo Jiedu formula (HZJD) attenuated gastric mucosal lesions in CAG model rats by remodeling the intestinal microbial community and its metabolite milieu [212]. Their study offers a compelling template for dissecting TCM mechanisms in CAG across interconnected microecological, metabolic, and host-response axes.

The convergence of systems biology and network pharmacology has enabled the decoding, at the molecular level, of the basis of integrative Chinese–Western treatment strategies for CAG. Weng et al. combined meta-analysis with network pharmacological modeling to evaluate TCM efficacy in CAG, pinpointing 90 shared drug-disease target genes; KEGG pathway enrichment and molecular docking further showed that naringenin, luteolin, and quercetin bind MAPK1 and MAPK3 with high affinity [213]. In contrast, Wang et al. mined 6253 traditional Chinese medicine electronic medical records, constructed a patient–symptom similarity network (PSN), and applied community detection algorithms to stratify patients with CAG into clinically coherent subgroups; network pharmacology then mapped syndrome-specific symptom profiles onto corresponding herbal prescriptions and their molecular targets, furnishing data-driven support for precision syndrome differentiation [214]. Together, these studies validate the internal logic linking syndrome classification, targeted herbal medication, and molecular mechanisms at the population scale and lay the groundwork for integrating microecological regulatory principles into precision combinatorial therapies for CAG.

Overall, accumulating preclinical and clinical evidence suggests that TCM formulae can modulate the gastric and intestinal microbiota while simultaneously reinforcing the gastric mucosal barrier and regulating inflammatory and immune responses through bioactive constituents such as naringenin, luteolin, and quercetin [215]. The robustness of this evidence, however, is currently limited by small trial sizes, heterogeneous study designs, and the near-absence of microbiome-specific primary endpoints in published clinical trials. Rigorously designed prospective trials incorporating standardized microbiome sampling and histological endpoints are needed to establish the microecological basis of integrative Chinese-Western strategies before they can be recommended as evidence-based interventions for CAG.

## 6. Summary and Future Perspectives: Key Findings and Limitations of Current Research

This review provides a systematic overview of the pivotal role of gastric microecological dysbiosis in CAG pathogenesis and the therapeutic potential of microecological agents. Available evidence indicates that the intragastric microbiota undergoes directional restructuring along the continuum from healthy mucosa through CAG to GC. Alpha diversity declines progressively, oral-derived taxa and facultatively anaerobic opportunistic pathobionts become enriched, and traditionally commensal genera are markedly depleted. This evolutionary pattern, characterized by commensal attrition, pathobiont expansion, and functional reprogramming of the microecological network, constitutes a critical ecological foundation for the neoplastic progression of CAG.

Microecologica0l interventions have demonstrated clear efficacy in augmenting *H. pylori* eradication, attenuating gastric mucosal inflammation, and alleviating clinical symptomatology. Whether probiotics can reverse or delay established CAG lesions and improve long-term outcomes remains uncertain because the available evidence is limited. A conceptual parallel exists in cancer immunotherapy research, where the gut microbiota has been proposed as a prerequisite threshold for therapeutic responsiveness rather than a mere efficacy enhancer, a framework that may help explain similar sources of variability in CAG [216]. The effects of strain specificity, dose, baseline microbia composition, host genetics, and habitual diet on intervention efficacy also require further investigation. Because gastric microecological dysbiosis represents a central component of CAG pathogenesis, microecology-directed strategies may offer new opportunities for both prevention and treatment. Notably, artificial intelligence and machine learning are increasingly being applied across two complementary dimensions of CAG research. In the diagnostic domain, deep learning models trained on endoscopic image datasets have demonstrated sensitivity and specificity exceeding 90% for CAG recognition, with performance comparable to or surpassing that of experienced endoscopists, and have further shown capacity for automated OLGA staging in prospective clinical cohorts [217,218]. In parallel, ML-based integration of microbiome sequencing data has enabled the construction of microbial feature panels capable of discriminating CAG and GC from healthy controls with high accuracy, offering a potential framework for non-invasive risk stratification along the Correa cascade [219]. Despite these advances, critical barriers including limited external validation, dataset heterogeneity, and the absence of prospective intervention trials constrain the current translational readiness of AI-guided microecological diagnostics and therapeutic decision-making in CAG. As multi-omics technologies, synthetic biology, artificial intelligence, and integrative Chinese-Western medical research continue to advance, a precision, individualized microecological diagnostic and therapeutic framework will assume an increasingly prominent role in CAG management and GC prevention, ultimately contributing to the reduction in the global GC burden.

Beyond these general directions, several unmet research needs warrant priority attention. Well-designed longitudinal cohort studies, rather than the predominantly cross-sectional designs currently available, are needed to clarify the temporal relationship between microbial restructuring and the progression of atrophic and metaplastic lesions, thereby helping to distinguish whether dysbiosis represents a cause or a consequence of mucosal injury. This temporal resolution would be strengthened by multi-center validation cohorts incorporating standardized sampling protocols, including consistent biopsy sites, processing procedures, and sequencing platforms, which may reduce methodological heterogeneity and improve cross-study comparability and biomarker robustness. Complementary integration of metagenomic and metabolomic approaches may further help link microbial compositional changes with functional metabolic outputs, allowing better discrimination between causally relevant and incidental alterations. Ultimately, interventional trials targeting premalignant lesions, with histological regression of atrophy or intestinal metaplasia as primary endpoints rather than symptomatic improvement or *H. pylori* eradication alone, will be required before microecological strategies can be more confidently translated into clinical management frameworks for CAG.

## Figures and Tables

**Table 3:** Representative Studies of Microbiome-Based Interventions in CAG and *H. pylori*–Associated Gastric Disease: In Vitro, Animal, and Human Evidence.

Intervention Type	Study	Population/Model	Intervention	Dosage and Duration	Primary Endpoint	Key Outcomes	Effect on *H. pylori*	Effect on Atrophy/IM
**In vitro Studies**								
Probiotics	**Song H (2019)** [174]	*H. pylori*–challenged AGS cells	*L. rhamnosus* GG, *L. acidophilus* LA5, *L. casei* DN-114001, *B. bifidum* BB12, *L. plantarum* 299v	1 × 10^8^–10^9^ CFU/mL; AGS cells pre-incubated with probiotics for 1 h, followed by addition of *H. pylori* (1 × 10^8^ CFU/mL) and co-culture for 6 h	*H. pylori* adhesion and growth inhibition; IL-8 suppression	Inhibition of *H. pylori* growth and adhesion; downregulation of IL-8 in epithelial cells	Adhesion inhibited; growth suppressed in vitro	N/A (in vitro model; no atrophy or IM endpoint)
Probiotics	**Yang YJ (2025)** **—in vitro arm** [177]	*H. pylori*–challenged GES-1 (human normal gastric epithelial) and AGS (human gastric adenocarcinoma) cells	*Lactobacillus acidophilus* + *Bifidobacterium lactis*	MOI 100; 4 h probiotic pre-treatment followed by 24 h *H. pylori* co-culture	Mechanistic pathway analysis: Wnt/β-catenin signaling, COX-2 expression, miRNA profiling	Downregulation of Wnt/β-catenin and COX-2 pathways; altered miRNA expression; provides mechanistic rationale for IM reversal observed in clinical arm	*H. pylori*–challenged model; downstream inflammatory and oncogenic pathway modulation assessed	Mechanistic basis for IM-associated pathway reversal demonstrated in vitro
**Animal Studies**								
Probiotics	**Yu YY (2023)** [175]	*H. pylori*–infected C57BL/6 mice	*Lactobacillus plantarum* ZFM4	Oral gavage: 1 × 10^9^ CFU/mL, 400 μL/day. Prophylactic regimen: 4-week probiotic treatment → 4-week *H. pylori* administration (every other day). Therapeutic regimen: 4-week *H. pylori* → 4-week probiotic treatment	*H. pylori* colonization load; inflammatory cytokines (IL-1β, IL-6, TNF-α); histopathology; mucosal barrier proteins	*H. pylori* load ↓; ↓ IL-1β, IL-6, TNF-α; improved histopathology scores; enhanced mucosal barrier protein expression	Colonization reduced (prophylactic administration more effective than therapeutic)	Histopathological scores improved; IM not specifically assessed
Postbiotics	**Yu J (2024)** [176]	*H. pylori*–infected female C57BL/6 mice (6 weeks, 16–18 g)	*Lacticaseibacillus paracasei* ZFM54 exopolysaccharide (EPS54)	2/4/8 mg/day (5/10/20 g/L), 400 μL/day, oral gavage, once daily; 2-week infection establishment + 4-week treatment	Gastric histopathology; inflammatory cytokine regulation; gastric microbiota composition	Histopathological improvement; ↓ inflammatory cytokines; gastric microbiota modulation	*H. pylori*–associated inflammation attenuated; eradication not an endpoint	Histopathological improvement; IM not specifically assessed
**Human Studies**								
Probiotics	**Yang YJ (2025)** **— clinical arm** [177]	RCT (*n* = 58; 32 probiotic vs. 26 control); *H. pylori*–eradicated patients with residual IM	*Lactobacillus acidophilus* + *Bifidobacterium lactis*, oral	Oral probiotic treatment; IM regression evaluated after 6 months of intervention	**Histological: IM regression**	IM regression observed; consistent with mechanistic findings from in vitro arm	Post-eradication cohort; independent eradication efficacy not assessed	IM regression demonstrated; preliminary finding from a single small RCT (*n* = 58); requires confirmation in larger, adequately powered trials
Probiotics	**Minoretti P (2024)** [180]	Open-label clinical trial; *H. pylori*–negative pilots with chronic non-atrophic gastritis	*Saccharomyces boulardii* (6 × 10^9^ CFU/day) + *Enterococcus faecium* (2 × 10^9^ CFU/day), compound probiotic capsule	Oral, twice daily (before meals); low dose: 1 capsule/dose; high dose: 2 capsules/dose; 4 weeks (28 days)	**Symptom improvement only (VAS gastric symptom score; GIQLI); no histological endpoint included**	↑ VAS symptom score; ↓ gastric discomfort rate; ↑ GIQLI quality-of-life score; well tolerated	N/A (*H. pylori*–negative population)	Not assessed; non-atrophic gastritis population; symptom improvement cannot be interpreted as evidence of atrophy or IM reversal

Primary Endpoint: For human studies, primary endpoints are explicitly specified to differentiate histological outcomes (atrophy or intestinal metaplasia regression) from symptomatic endpoints. Symptom improvement alone does not constitute evidence of mucosal atrophy or metaplasia reversal. Arrow notation: ↑ indicates an increase; ↓ indicates a decrease. Note: No CAG-specific interventional studies were identified for prebiotics or synbiotics within the search period of this review; evidence for these categories in CAG remains limited to mechanistic and observational data. Abbreviations: CAG, chronic atrophic gastritis; IM, intestinal metaplasia; *H. pylori*, *Helicobacter pylori*; RCT, randomized controlled trial; MOI, multiplicity of infection; CFU, colony-forming unit; VAS, visual analog scale; GIQLI, Gastrointestinal Quality of Life Index; N/A, not applicable.

**Table 4:** Comparison of Probiotics, Prebiotics, Synbiotics, and Postbiotics in CAG: Characteristics, Evidence Levels, and Potential Clinical Applications.

	Probiotics	Prebiotics	Synbiotics	Postbiotics
Representative Components	*Lactobacillus*, *Bifidobacterium*, *S. boulardii*, *Streptococcus thermophilus* [185]	FOS, GOS, inulin, XOS, resistant starch [184]	Live microorganisms: *Lactobacillus*, *Bifidobacterium*, *S. boulardii*; *Substrates*: FOS, GOS, inulin, XOS [193]	Inactivated bacteria: Heat-killed *Lactobacillus*; Metabolites: SCFAs, bacteriocins, peptides [194]
Stability	Low: Requires refrigeration (2–8 °C), has a relatively short shelf-life, and is sensitive to heat and acid [185]	High: Stable at room temperature, not dependent on bacterial survival [183]	Low: Requires refrigeration (2–8 °C), has a relatively short shelf life, and is sensitive to heat and acids [195].	High: Can be stored at room temperature, has a long shelf-life (up to 2 years), and is heat- and acid-resistant [188].
Safety	Caution required: Potential Risk in immunocompromised individuals, potential gene transfer, and possible interference with neonatal gut colonization [185]	High: Generally safe, no risk of live bacterial infection, suitable for all populations [183]	Caution required: potential risk in immunocompromised individuals, antibiotic resistance gene transfer, and possible interference with neonatal gut colonization [195].	High: No risk of live bacterial infection, no bacterial translocation, and suitable for vulnerable populations [191].
Mode of Action	Colonization-dependent microbiota modulation [185]	Selective stimulation of beneficial microbes [184]	Combined microbial and substrate effects	Direct delivery of bioactive molecules [194].
Gastric Mucosal Protection	Barrier enhancement, pathogen inhibition, immune regulation [196,197,198]	SCFA-mediated barrier support and microbiota modulation	Combined barrier-protective and immunomodulatory effects [185,196]	Direct anti-inflammatory and epithelial repair effects
Evidence in CAG	Supported by multiple RCTs and meta-analyses; represents the strongest CAG-specific clinical evidence base among the four categories [168,169,190]	Primarily mechanistic and observational studies; CAG-specific RCT evidence is currently limited	Emerging evidence, largely extrapolated from probiotic trials; synbiotic-specific RCT data in CAG are limited	Mainly preclinical studies; human CAG-specific clinical evidence is currently lacking
Effect on *H. pylori* Eradication	Adjunct supplementation increases eradication rates by 10–15% and reduces antibiotic-associated adverse effects [190]	Indirect support through promotion of beneficial microbiota; no direct evidence of improved eradication rates in clinical studies	Potential additive benefit from combined probiotic and prebiotic components; direct CAG-specific evidence is limited	Not established
Effect on Gastric Inflammation	Attenuation of proinflammatory cytokines (IL-1β, IL-6, TNF-α); NLRP3 inflammasome suppression via PINK1/Parkin-mediated mitophagy [199]; immune reprogramming via tolerogenic DC induction (murine model) [200]	SCFA-mediated indirect anti-inflammatory effects; no direct gastric-specific clinical evidence in CAG	Combined anti-inflammatory potential from probiotic and prebiotic components; limited CAG-specific data	Preclinical evidence of anti-inflammatory effects in a murine *H. pylori* model using exopolysaccharide (EPS54) [176]; human CAG-specific clinical data are currently lacking
Effect on Gastric Atrophy	Limited evidence of direct reversal; may help delay further progression when used as adjunct to *H. pylori* eradication therapy	Not established	Not established	Not established
Effect on Intestinal Metaplasia	Preliminary clinical evidence of IM regression reported in one small RCT (*n* = 58; 6-month intervention) [177]; findings require confirmation in larger, adequately powered trials	Not established	Not established	Not established
Advantages	Sustained colonization-mediated effects; microbiota and immune modulation; extensive clinical validation.	Safe, stable, and suitable for long-term microbiota support.	Synergistic microbiota modulation with combined probiotic and prebiotic benefits.	Highly safe and stable; rapid bioactivity; suitable for use with antibiotics and in vulnerable populations.
Disadvantages	Colonization-dependent; storage-sensitive; potential safety concerns in immunocompromised hosts.	Indirect mechanism and delayed onset; requires continuous intake.	Dependent on probiotic viability and colonization; similar safety considerations as probiotics.	Limited clinical evidence; may require repeated administration; higher manufacturing costs.
Potential Application in CAG	Adjunct to *H. pylori* eradication and symptom management	Long-term microbiota support	Restoration of microbial homeostasis following eradication therapy	Potential anti-inflammatory strategy for vulnerable populations

Entries marked “Not established” indicate that no CAG-specific clinical or preclinical evidence addressing that outcome was identified within the search scope of this review, rather than evidence of an absence of effect. Several applications listed for prebiotics, synbiotics, and postbiotics, particularly under “Potential Application in CAG,” are extrapolated from probiotic trials, other gastrointestinal conditions, or preclinical models rather than supported by CAG-specific clinical evidence; readers should interpret these entries in conjunction with the corresponding “Evidence in CAG” row above. Abbreviations: CAG, chronic atrophic gastritis; *H. pylori*, *Helicobacter pylori*; IM, intestinal metaplasia; RCT, randomized controlled trial; FOS, fructo-oligosaccharides; GOS, galacto-oligosaccharides; XOS, xylo-oligosaccharides; *S. boulardii*, *Saccharomyces boulardii*; SCFAs, short-chain fatty acids; IL-1β, interleukin-1β; IL-6, interleukin-6; TNF-α, tumor necrosis factor-α; NLRP3, NOD-like receptor family pyrin domain-containing 3; DC, dendritic cell; EPS54, exopolysaccharide 54.

## Data Availability

No new data were created or analyzed in this study.

## Abbreviations

**Abbreviation**	**Full Term**
AAG	Autoimmune atrophic gastritis
ADAM17	A disintegrin and metalloproteinase 17
AG	Atrophic gastritis
CAG	Chronic atrophic gastritis
DGR	Duodenogastric reflux
EBV	Epstein–Barr virus
EGFR	Epidermal growth factor receptor
EVs	Extracellular vesicles
FAO	Fatty acid oxidation
FOS	Fructo-oligosaccharides
FXR	Farnesoid X receptor
GC	Gastric cancer
GOS	Galacto-oligosaccharides
GPR	G protein-coupled receptor
*H*. *pylori*	*Helicobacter pylori*
HB-EGF	Heparin-binding EGF-like growth factor
HDACs	Histone deacetylases
HIF-1α	Hypoxia-inducible factor 1-alpha
ILC2s	Group 2 innate lymphoid cells
IM	Intestinal metaplasia
LPS	Lipopolysaccharide
MGV	Metagenomic Gut Virus catalog
MOI	Multiplicity of infection
mTOR	Mechanistic target of rapamycin
NF-κB	Nuclear factor kappa B
NLRP3	NOD-like receptor family pyrin domain-containing 3
OMVs	Outer membrane vesicles
OXPHOS	Oxidative phosphorylation
PPI	Proton pump inhibitor
PRRs	Pattern recognition receptors
RCT	Randomized controlled trial
RNS	Reactive nitrogen species
ROS	Reactive oxygen species
SCFAs	Short-chain fatty acids
SIBO	Small intestinal bacterial overgrowth
SPEM	Spasmolytic polypeptide-expressing metaplasia
TCA cycle	Tricarboxylic acid cycle
TCGA	The Cancer Genome Atlas
TCM	Traditional Chinese medicine
TFF2	Trefoil factor family 2
TGR5	Takeda G protein-coupled receptor 5
Th17	T helper 17 cells
TLRs	Toll-like receptors
Tregs	Regulatory T cells
XOS	Xylo-oligosaccharides
ZO-1	Zonula occludens-1
PAMPs	Pathogen-associated molecular patterns
